# qDNAmod: a statistical model-based tool to reveal intercellular heterogeneity of DNA modification from SMRT sequencing data

**DOI:** 10.1093/nar/gku1097

**Published:** 2014-11-17

**Authors:** Zhixing Feng, Jing Li, Jing-Ren Zhang, Xuegong Zhang

**Affiliations:** 1 MOE Key Lab of Bioinformatics, Bioinformatics Division, TNLIST and Department of Automation, Tsinghua University, Beijing 100084, China; 2 Center for Infectious Disease Research, School of Medicine, Tsinghua University, Beijing 100084, China; 3 Collaborative Innovation Center for Biotherapy, Tsinghua University, Beijing 100084, China; 4 Collaborative Innovation Center for Biotherapy, State Key Laboratory of Biotherapy and Cancer Center, West China Hospital, West China Medical School, Sichuan University, Chengdu, China

## Abstract

In an isogenic cell population, phenotypic heterogeneity among individual cells is common and critical for survival of the population under different environment conditions. DNA modification is an important epigenetic factor that can regulate phenotypic heterogeneity. The single molecule real-time (SMRT) sequencing technology provides a unique platform for detecting a wide range of DNA modifications, including N6-methyladenine (6-mA), N4-methylcytosine (4-mC) and 5-methylcytosine (5-mC). Here we present qDNAmod, a novel bioinformatic tool for genome-wide quantitative profiling of intercellular heterogeneity of DNA modification from SMRT sequencing data. It is capable of estimating proportion of isogenic haploid cells, in which the same loci of the genome are differentially modified. We tested the reliability of qDNAmod with the SMRT sequencing data of *Streptococcus pneumoniae* strain ST556. qDNAmod detected extensive intercellular heterogeneity of DNA methylation (6-mA) in a clonal population of ST556. Subsequent biochemical analyses revealed that the recognition sequences of two type I restriction–modification (R-M) systems are responsible for the intercellular heterogeneity of DNA methylation initially identified by qDNAmod. qDNAmod thus represents a valuable tool for studying intercellular phenotypic heterogeneity from genome-wide DNA modification.

## INTRODUCTION

DNA modification is important for many important functions in both prokaryotic and eukaryotic organisms, such as gene regulation, cellular differentiation and DNA repair (1,2). Common genomic modifications include 5-methylcytosine (5-mC), N4-methylcytosine (4-mC) and N6-methyladenine (6-mA). DNA modification status of a given genome locus can differ from cell to cell in a population, which is referred to as intercellular heterogeneity of DNA modification (1,2). Intercellular heterogeneity of DNA modification is a major epigenetic cause of phenotypic heterogeneity in many eukaryotic and prokaryotic organisms. In bacteria, DNA modification-driven phenotypic heterogeneity is best studied in *Escherichia coli*, particularly the ON/OFF reversible bistability or phase variation of the pyelonephritis-associated pili (Pap) (3,4) and antigen 43 (Ag43) (5,6). The expression of Pap and Ag43 is regulated by the methylation status of multiple GATC sequences (the recognition motif for Dam methyltransferase) in the promoter regions of *pap* and *agn43* loci. Differential methylation of the GATC sequences by Dam from cell to cell leads to ON/OFF expression of Pap and Ag43 in a clonal population, so-called phase variation (7). A more recently discovered example for intercellular heterogeneity of DNA modification in bacteria is phasevarions (phase variable regulons) in human pathogens *Haemophilus influenzae* (8,9), *Neisseria gonorrhoeae* (10), *Neisseria meningitidis* (10), *Helicobacter pylori* (11) and *Moraxella catarrhalis* (12). Phasevarions are driven by mutations in the repeat sequences encoding the methyltransferases of the type III restriction–modification (R-M) systems (13). The ON/OFF phase variation in the activity of the methyltransferases among different cells leads to intercellular heterogeneity in DNA modification, gene expression and bacterial responses to stressful conditions (9,13). Therefore, genome-wide profiling intercellular heterogeneity of DNA modification can provide valuable information for understanding phenotypic variation.

Traditionally, genome-wide DNA modification detection was restricted to 5-mC due to the hurdle of detection technology (14). The emerging single molecule real-time (SMRT) sequencing technology provides a novel platform for genome-wide detecting a wide range of DNA modifications, including 5-mC, 4-mC, 6-mA and DNA damage at single molecular level (15). SMRT sequencing has been successfully applied to 5-hydroxymethylcytosine (5-hmC) profiling (16), methylome profiling in a number of bacteria (17–20) and DNA damage analysis (21,22). SMRT sequencing also provides a powerful way for quantitative detection of DNA modification heterogeneity. However, the unique data type of SMRT sequencing posts new challenges for the existing data analysis methods. The main difficulty is that the signal (inter pulse duration (IPD) distribution, see the definition in empirical Bayes mixture model) of kinetic variant bases is usually unknown, and it has to be learnt from the data. One idea to tackle this issue is fitting a standard two-component mixture model (see the Supplemental Methods for details) for the *k*th locus by using its IPDs, $\mathbf {\it y_k}$, so that IPD distribution of kinetic variant bases can be learnt from $\mathbf {\it y_k}$. This method is accurate when there are a reasonable number of kinetic variant bases covering the *k*th locus. However, it is inaccurate when there is no kinetic variant base covering the *k*th locus because $\mathbf {\it y_k}$ does not provide sufficient information of IPD distribution of a kinetic variant base in this case. Any other methods that use only $\mathbf {\it y_k}$ may not perform well due to the same reason.

To help solve this problem, we developed a bioinformatic tool, qDNAmod, on the basis of an empirical Bayes mixture model. This model uses whole genome data to learn IPD distribution of kinetic variant bases from bases that are likely to be kinetic variant. The empirical Bayes mixture model employed by qDNAmod is a general method, which is able to quantitatively detect intercellular heterogeneity of known modification types, including 5-mC, 4-mC and 6-mA, as well as novel modification types. qDNAmod was first tested with the simulated data and the SMRT sequencing data of *Streptococcus pneumoniae* strain ST556, a multidrug-resistant pathogen (23). qDNAmod analysis predicted extensive intercellular heterogeneity of DNA methylation in the genome of a clonal *S. pneumoniae* population, implying the existence of multiple subpopulations. Further functional experiments verified the incomplete methylation for the type I R-M recognition motifs predicted by qDNAmod. To the best of our knowledge, qDNAmod is the first statistical model-based tool for detecting intercellular heterogeneity of DNA modification from SMRT sequencing data, and the result reported by qDNAmod on the *S. pneumoniae* ST556 data is the first genome-wide profile of intercellular adenine methylation heterogeneity in bacteria.

## MATERIALS AND METHODS

### Simulated data sets construction

The number of bases covering each locus, denoted by *m*_*k*_, the number of times each base being sequenced, denoted by *n*_*ki*_, and the effect size, denoted by *d*_*k*_ (see the definition in Equation (5)), are three major factors that affect performance of DNA modification proportion estimation algorithms. We constructed six simulated data sets with different *m*_*k*_, *n*_*ki*_ and *d*_*k*_ to evaluate performance of qDNAmod (Table 1). In the simulated data sets, the genome size was set to be 2 145 902 bp, which is the same with the genome size of *S. pneumoniae* ST556. For the *k*th locus, its IPDs (see details in the empirical Bayes mixture model of the Results section) were generated from the distribution defined by Equations (1) and (2). In Data 1 and Data 2 in Table 1, *m*_*k*_ and *n*_*k*_ were sampled from their empirical distribution estimated from the real data (*S. pneumoniae* whole genome amplification (WGA) in Table 2) shown in Supplementary Figure S1 (average *m*_*k*_ is 41.5 and average *n*_*ki*_ is 5.0). In Data 3 and Data 4 in Table 1, *m*_*k*_ was set to be five times of the *m*_*k*_ used in Data 1 and Data 2 and *n*_*ki*_ was set to be 1. In Data 5 and Data 6 in Table 1, *m*_*k*_ was set to be 1/8 of the *m*_*k*_ used in Data 1 and Data 2 and *n*_*ki*_ was set to be eight times of the *n*_*ki*_ used in Data 1 and Data 2. We used 10 000 modified loci in each data set and evenly divided them into 10 groups with different modification proportions (*p*_*k*_ = 0.1, 0.2, ..., 1). *p*_*k*_ of the other loci were set to 0. *μ*_*k*0_ and σ_*k*0_ were estimated from the real data (*S. pneumoniae* WGA in Table 2). σ_*k*1_ was set to be equal to σ_*k*0_ and *μ*_*k*1_ was set to be *μ*_*k*1_ = *μ*_*k*0_ + *d*_*k*_. In the simulated data sets, a modified base can only affect its own IPD distribution. A kinetic variant base is a modified base in the simulated data sets.

**Table 1.** Simulated data sets of SMRT sequencing

Sample	Average *m*_*k*_	Average *n*_*ki*_	*d*_*k*_
Data 1	41.5	5.0	2.5σ_*k*0_
Data 2	41.5	5.0	1.25σ_*k*0_
Data 3	207.5	1.0	2.5σ_*k*0_
Data 4	207.5	1.0	1.25σ_*k*0_
Data 5	5.2	40.0	2.5σ_*k*0_
Data 6	5.2	40.0	1.25σ_*k*0_

**Table 2.** SMRT sequencing data

Sample	Coverage per strand	Average DNA fragment size	Average read length	NCBI SRA ID
*S. pneumoniae* OD_620_ = 0.08	342x	206 bp	3466 bp	SRX735344
*S. pneumoniae* OD_620_ = 0.5	247x	200 bp	3754 bp	SRX735345
*S. pneumoniae* OD_620_ = 0.8	313x	200 bp	3394 bp	SRX735346
*S. pneumoniae* WGA	231x	201 bp	3560 bp	SRX735347

### Bacterial strains and chemical reagents

We applied our model for genome-wide profiling heterogeneity of DNA modification in *S. pneumoniae* strain ST556. ST556 is a serotype 19F multidrug-resistant isolate from an otitis media patient (23). The complete genome sequence of ST556 has been determined (24). The bacterial strains used in this study are listed in Supplementary Table S1. Pneumococci were grown in Todd-Hewitt broth containing 0.5% yeast extract (THY) or on tryptic soy agar plates containing 3% (v/v) sheep blood at 37°C with 5% CO_2_ as described (23). When necessary, kanamycin (400 μg/ml) or chloramphenicol (4 μg/ml) was added in the broth or agar media for selection purposes. *E. coli* strains were grown in Luria-Bertani (LB) broth or on LB agar plates in the presence or absence of ampicillin (100 μg/ml). *E. coli* strain ER2796 was a gift from Dr Richard J. Roberts from New England Biolabs (20). All chemicals used in this work were obtained from Sigma-Aldrich (St. Louis, MO, USA) unless otherwise stated. All restriction enzymes were purchased from New England Biolabs (NEB, Ipswich, MA, USA). The Phusion high-fidelity DNA polymerase (NEB, Ipswich, MA, USA) was used for all polymerase chain reactions (PCRs).

### Preparation of *S. pneumoniae* genomic DNA

The genomic DNA of *S. pneumoniae* ST556 was prepared for SMRT sequencing as described (25). Briefly, a single colony of *S. pneumoniae* ST556 was picked from a freshly streaked plate and inoculated into THY broth. The pneumococci were grown to an OD (optical density) of 0.5 at a wavelength of 620 nm as a seed culture. A subculture was then prepared by mixing 100 ml of the seed culture with 900 ml of fresh THY broth preheated to 37°C. To purify genomic DNA, a fraction of the subculture was removed at three bacterial densities (OD_620_= 0.08, OD_620_= 0.5 and OD_620_= 0.8). The genomic DNA was extracted by using the DNeasy Blood & Tissue Kit (Qiagen, Hilden, Germany) (25). WGA of *S. pneumoniae* ST556 was carried out by PCR with the phi29 DNA polymerase (NEB, Ipswich, MA, USA) as described (26). The resulting DNA samples were analyzed by agarose gel electrophoresis and NanoDrop^TM^ 2000 (Thermo Scientific, Waltham, MA, USA).

### SMRT sequencing and data pre-processing

The SMRT sequencing was performed in the W. M. Keck Foundation Biotechnology Resource Laboratory at Yale University. SMRTbell template libraries were prepared using standard SMRTbell template preparation protocols on the PacBio RS (Pacific Biosciences, Menlo Park, CA, USA) as described (27).

Because IPDs around insertions, deletions and mismatches are difficult to define on the basis of the SMRT sequencing principle (28), we removed them before further analysis (illustrated in Supplementary Figure S2). The IPDs were then Box–Cox transformed as described (29).

### Construction of R-M system mutants

The R-M system mutants of *S. pneumoniae* strain ST556 were constructed by replacing the R-M genes with the Janus cassette as described (30). The up- and downstream regions of MYY0570-MYY0572 (type I R-M system) were amplified by PCR from genomic DNA of *S. pneumoniae* ST556 using primer pairs Pr6929/Pr6930 and Pr6931/Pr6932, respectively. Primer pairs Pr6941/Pr6942 and Pr6943/Pr6944 were used to amplify the flanking regions of MYY0859-MYY0860 (type II R-M system). The Janus cassette was amplified with primers Pr1097 and Pr1098 from strain ST588 (30). All mutations were confirmed by PCR amplification and DNA sequencing. The transformants were selected with kanamycin. All primers used in this study are described in Supplementary Table S3.

### Restriction enzyme digestion

The plasmids used in this study are listed in Supplementary Table S2. The sequence specificities of the R-M systems in *S. pneumoniae* strain ST556 were determined by restriction digestion with methylation-sensitive restriction enzymes as described (20). Based on the genome annotation of *S. pneumoniae* ST556 (accession CP003357), the genes encoding putative type I R-M *hsdS* and *hsdM* were PCR amplified from *S. pneumoniae* ST556 genomic DNA using primer pairs Pr6993/Pr6994 (MYY0570-MYY0571) and Pr7094/Pr7095 (MYY1311-MYY1312). Similarly, the type II R-M MTase (MYY0859) was amplified with primers Pr6991/Pr6992. The PCR products were cloned in the PstI-BamHI sites of the pRRS plasmid in *E. coli* strain ER2796 as described (20), resulting in pTH4834. pRRS was a gift from Dr Richard J. Roberts from NEB. Based on the SMRT sequencing results, desirable methylation motifs in the *S. pneumoniae* ST556 genome were added to the 3′ ends of the recombinant plasmids by the *in vivo* recombination method as illustrated in Figure 4 (31). Briefly, the recombinant plasmids were PCR amplified from the plasmids with the primers pairs Pr7389/Pr7393, Pr7390/Pr7395 and Pr7102/Pr7109, and transformed into *E. coli* ER2796, resulting in plasmids pTH4832, pTH4833 and pTH4835. These primers contained the methylation motifs and additional sequences. Expression of the insert sequences was driven by an existing promoter in pRRS (20). The methylation motifs include 5′-AAG(N)_8_TTTG-3′, 5′-AAG(N)_8_TTCG-3′, 5′-TGA(N)_7_TATC-3′ and 5′-TCTAGA-3′, which were among those revealed by the SMRT sequencing results. The specific sequences representing these motifs are shown in Figure 3A. Plasmids containing inactive methyltransferases were generated by inserting an adenine nucleotide immediately following the ATG start codons of the wild-type methyltransferase genes by PCR amplification of the plasmids containing the wild-type methyltransferase genes. Specifically, plasmids pTH4832, pTH4833, pTH4834 and pTH4835 were amplified with primer pairs Pr7742/Pr7743, Pr7742/Pr7743, Pr7744/Pr7745 and Pr7746/Pr7747, respectively, resulting in frame-shifted methyltransferase-encoding genes in plasmids pTH4836 (MYY0571), pTH4837 (MYY0571), pTH4838 (MYY0859) and pTH4839 (MYY1312).

To detect methylation status of the sequence motifs, the recombinant plasmids were extracted from *E. coli* cultures. As unmethylated DNA controls, each plasmid was PCR amplified using primer pairs Pr7797/Pr7798 (pTH4832), Pr7797/Pr7798 (pTH4833), Pr7799/Pr7800 (pTH4834) and Pr7801/Pr7802 (pTH4835). The plasmids and their linear PCR products were digested with PstI and a cognate methylation-sensitive restriction enzyme as follows: ScaI for 5′-AAG(N)_8_TTTG-3′ (pTH4832 and pTH4836) and 5′-AAG(N)_8_TTCG-3′ (pTH4833 and pTH4837), XbaI for 5′-TCTAGA-3′ (pTH4834 and pTH4838) and SpeI for 5′-TGA(N)_7_TATC-3′ (pTH4835 and pTH4839). Restriction digestion reactions were performed according to the supplier's instructions. Digested DNA samples were separated by agarose gel electrophoresis, stained with GoldView^TM^ (NEB, Ipswich, MA, USA) and visualized with the ChemiDoc^TM^ XRS Imager (Bio-Rad, Hercules, CA, USA).

### Southern blotting

DNA methylation status in *S. pneumoniae* ST556 was detected by restriction digestion and Southern blotting as described (30). Briefly, the sequence motifs were added to the 3′ end of the chloramphenicol acetyltransferase gene in plasmid pST393 by PCR amplification using the following primer pairs: Pr7773/Pr7774 for 5′-**A****AG**TACTTCGG**TTTG**-3′, Pr7775/Pr7776 for 5′-**A****AG**TACTTTTT**TTTG**-3′, Pr7777/Pr7778 for 5′-**A****AG**TACTTGAG**TTCG**-3′, Pr7779/Pr7780 for 5′-**A****AG**TACTTTTT**TTCG**-3′ and Pr7771/Pr7772 for 5′-TCTAGA-3′ (Figure 5). pST393, a suicide plasmid of *S. pneumoniae*, contained a 248-bp insert representing *hk06* of *S. pneumoniae* (32). These PCR products were circularized by the *in vivo* recombination method in *E. coli* DH5α (31), resulting in recombinant plasmids pTH4994, pTH4995, pTH4996, pTH4997 and pTH4998. The plasmids were transformed in *S. pneumoniae* ST556 or isogenic mutants by natural transformation as described (30). For Southern hybridization, genomic DNA was extracted from *S. pneumoniae* ST556 when the bacteria were cultured to OD_620_ of 0.5 as described above. DNA samples were digested with methylation-sensitive restriction enzymes corresponding to individual motifs and additional enzyme when necessary as described above. Restriction-digested DNA fragments were separated by agarose gel electrophoresis and transferred to nylon membranes (GE Healthcare Lifescience, Pittsburgh, PA, USA) with 0.4-N NaOH as described (30). The DIG (digoxigenin)-dUTP (deoxyuridine triphosphate)-labeled probe was prepared by the PCR DIG Probe Synthesis Kit (Roche, Basel, Switzerland) as described in the supplier's instructions using primers Pr7653 and Pr7654. The location of the probe is indicated in Figure 5A. The DNA blots were hybridized with the probe; DNA fragments were detected by the DIG Luminescent Detection Kit (Roche, Basel, Switzerland) and visualized by the ChemiDoc^TM^ XRS Imager (Bio-Rad) as described previously (30).

## RESULTS

### Empirical Bayes mixture model

**Figure 1.**
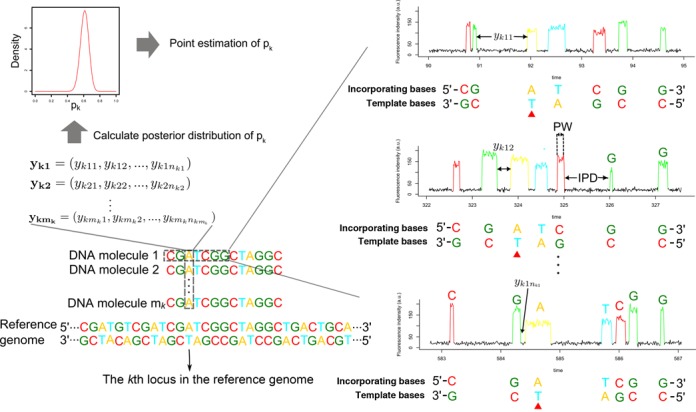
Quantitative detection of DNA modification heterogeneity. There are *m*_*k*_ bases covering the *k*th locus, and they are sequenced *n*_*k*1_, *n*_*k*2_, ... and $n_{k{m_k}}$ times, respectively. The ‘T’ at the *k*th locus in the reference genome has an IPD vector, denoted by $\mathbf {{\it y}_{{\it k}1}}$, $\mathbf {{\it y}_{{\it k}2}}$,..., $\mathbf {{\it y}_{\it km_k}}$. Given the IPD vector, the posterior distribution of proportion of kinetic variant bases covering the *k*th locus, *p*_*k*_, can be estimated by qDNAmod. A point estimation of *p*_*k*_ can be inferred from its posterior distribution.

SMRT sequencing data contain identity of each base (G, A, T or C) of the sequenced DNA molecules as well as the DNA polymerase kinetic in real time. DNA polymerase kinetic is measured by IPD and pulse width (PW) (Figure 1). It has been shown that IPD is highly sensitive to DNA modifications, and modification of a base may change IPD distribution of its about 10 flanking bases (15,17,29). SMRT sequencing adopts a sequencing-by-synthesis strategy and reports reverse complementary sequences of sequenced molecules. In this study, we refer to the sequences reported by SMRT sequencing as incorporating bases and to the sequences of the sequenced molecules as template bases. IPD of a base is defined as the IPD between the incorporating base complementary to it and the first upstream base of the incorporating base (Figure 1). A base whose IPD distribution is changed due to DNA modification is called kinetic variant base, otherwise called normal base. SMRT sequencing adopts a circular consensus sequencing strategy, which allows a base to be sequenced multiple times so that a base can have multiple IPDs (27). By comparing a base's IPD vector to the IPD distribution of a normal base with the same sequence context, it can be inferred whether the base is a kinetic variant (15,29). As there is no need for DNA amplification in SMRT sequencing, for haploid cells, different bases covering the same locus in SMRT sequencing data come from different cells if the original DNA sample is not amplified in the sample preparation step. Thus, proportion of cells whose DNA molecule is modified at a given genome locus is equal to proportion of modified bases covering that locus. We refer to proportion of cells whose DNA molecule is modified as DNA modification proportion for short. The aim of our model is to estimate DNA modification proportion at each genome locus.

Formally, as shown in Figure 1, there are *m*_*k*_ DNA molecules mapped to the *k*th locus in the reference genome, and the *i*th molecule is sequenced *n*_*ki*_ times. Box–Cox transformed IPDs of the *i*th base covering the *k*th locus are denoted by $\mathbf {\it y_{ki}}=(y_{ki1},y_{ki2},...,y_{kin_{ki}})$ and it has been shown that a Box–Cox transformed IPD follows a normal distribution approximately (29). (Hereafter, for simplicity, we refer to a Box–Cox transformed IPD as an IPD.) Denoting proportion of kinetic variant bases by *p*_*k*_, $\mathbf {\it y_{ki}}$ follows a normal mixture distribution, whose probability density function is

(1) \begin{eqnarray*} && f(\mathbf {\it y_{ki}}|\mu _{k0},\sigma _{k0}^2,\mu _{k1},\sigma _{k1}^2,p_k) \nonumber \\ &=& (1-p_k)\cdot N(\mathbf {\it y_{ki}}|\mu _{k0},\sigma _{k0}^2) + p_k\cdot N(\mathbf {\it y_{ki}}|\mu _{k1},\sigma _{k1}^2) \nonumber \\ &=& (1{-}p_k)\cdot \prod _{j=1}^{n_{ki}}N(y_{kij}|\mu _{k0},\sigma _{k0}^2){+}p_k\cdot \prod _{j=1}^{n_{ki}}N(y_{kij}|\mu _{k1},\sigma _{k1}^2), \nonumber \\ \end{eqnarray*}

where IPD of the base follows a normal distribution whose mean is *μ*_*k*0_ and variance is $\sigma _{k0}^2$, denoted by $N(\cdot |\mu _{k0},\sigma _{k0}^2)$, if it is normal, or follows a normal distribution whose mean is *μ*_*k*1_ and variance is $\sigma _{k1}^2$, denoted by $N(\cdot |\mu _{k1},\sigma _{k1}^2)$, if it is kinetic variant. Assuming IPDs from different molecules are independent, joint probability density function of all *m*_*k*_ bases is

(2) \begin{eqnarray*} &&f(\mathbf {\it y_k}|\mu _{k0},\sigma _{k0}^2,\mu _{k1},\sigma _{k1}^2,p_k)\nonumber \\ &=&\prod _{i=1}^{m_k}f(\mathbf {\it y_{ki}}|\mu _{k0},\sigma _{k0}^2,\mu _{k1},\sigma _{k1}^2,p_k), \end{eqnarray*}

where $\mathbf {{\it y}_{\it k}}=(\mathbf {{\it y}_{{\it k}1}},\mathbf {{\it y}_{{\it k}2}},...,\mathbf {{\it y}_{\it km_k}})$. Parameters *μ*_*k*0_ and $\sigma _{k0}^2$ can be predetermined by whole genome amplified (WGA) data. WGA data are SMRT sequencing data of a whole genome amplified sample whose DNA modifications have been removed. Assuming there are $m_k^c$ DNA molecules covering the *k*th locus in the WGA data and the *i*th base's IPD vector is denoted by $\mathbf {\it y_{ki}^c}=(y_{ki1}^c,y_{ki2}^c,...,y_{kin_{ki}^c}^c)$, $(\mu _{k0},\sigma _{k0}^2)$ can be estimated by mean and variance of IPDs of all $m_k^c$ bases, which are

(3) \begin{eqnarray*} \hat{\mu }_{k0} &=& \frac{1}{\sum _{i=1}^{m_k^c}n_{ki}^c}\sum _{i=1}^{m_k^c}\sum _{j=1}^{n_{ki}^c} y_{kij}^c \end{eqnarray*}

(4) \begin{eqnarray*} \hat{\sigma }_{k0}^2 &=& \frac{1}{\sum _{i=1}^{m_k^c}n_{ki}^c-1}\sum _{i=1}^{m_k^c}\sum _{j=1}^{n_{ki}^c}(y_{kij}^c-\hat{\mu }_{k0})^2 .\end{eqnarray*}

Assuming DNA modification does not affect variance ($\sigma _{k0}^2=\sigma _{k1}^2$), there are two parameters *μ*_*k*1_ and *p*_*k*_ in Equation (1) to be estimated from data $\mathbf {\it y_k}$. Intuitively, information about *μ*_*k*1_ is contained in IPDs of kinetic variant bases and estimation of *p*_*k*_ relies on *μ*_*k*1_. However, if the *k*th locus is not covered by any kinetic variant base, information about *μ*_*k*1_ is not available in $\mathbf {\it y_k}$, and estimating *p*_*k*_ based on $\mathbf {\it y_k}$ will lead to a very high error rate. To resolve this, we propose an empirical Bayes mixture model, which can make the *k*th locus borrow information from other loci covered by kinetic variant bases to estimate *μ*_*k*1_.

From a Bayesian point of view, there are two sources of information to estimate parameters of interest: one is the data and the other one is prior distribution of the parameters. If the *k*th locus is not covered by any kinetic variant bases, data $\mathbf {\it y_{k}}$ do not provide information about *μ*_*k*1_, the only way to estimate *μ*_*k*1_ is through an informative prior distribution of *μ*_*k*1_, whose probability density function is denoted by *f*(*μ*_*k*1_). We estimated *f*(*μ*_*k*1_) by leveraging genome-wide DNA polymerase kinetic information, $\mathbf {{\it y}_{1}},\mathbf {{\it y}_{2}},...,\mathbf {\it y_{N}}$, where *N* is the genome size. Besides DNA modification, *μ*_*k*1_ is also affected by sequence context (29). Because the impact of DNA modification on the Box–Cox transformed IPD is largely a shift of mean (Supplementary Figure S6), to reduce the impact of sequence context, we used genome-wide data to estimate *g*(*d*_*k*_), the probability density function of modification effect size

(5) \begin{eqnarray*} d_{k}&=&\mu _{k1}-\mu _{k0} \end{eqnarray*}

first and obtained the probability density function of *μ*_*k*1_ by shifting. Formally, *f*(*μ*_*k*1_) = *g*(*μ*_*k*1_ − *μ*_*k*0_) (details are given in the Supplemental Methods). Briefly, we evaluated the strength of evidence supporting each base is kinetic variant by comparing its IPD vector to the control data first and leveraged IPD information of bases that have modest or strong evidence to be kinetic variant to estimate *f*(*μ*_*k*1_) by a weighted histogram approach.

We assumed that prior distribution of *p*_*k*_ is non-informative. Formally,

(6) \begin{eqnarray*} f(p_k) &=& U(0,1), \end{eqnarray*}

where *U*(0, 1) is the probability density function of uniform distribution between 0 and 1. By using the estimated prior distribution of *μ*_*k*1_, $\hat{f}(\mu _{k1})$, we calculated the posterior distribution of (*p*_*k*_, *μ*_*k*1_) by variational inference and used maximum *a posteriori* as the point estimator of *p*_*k*_ (details are given in the Supplemental Methods).

It should be noted that a kinetic variant base is not necessarily modified, because a modified base can affect IPD distribution of its about 10 flanking bases (15,17,29). Generally, resolution of DNA modification detection is not single base, although the exact modified base can be pinpointed empirically for some known modification type, such as 6-mA, according to their featured IPD profiles (15). Thus, we used a sliding window approach to estimate the DNA modification proportion of the modified locus in the window. By assuming that there is only one modified locus in the window, we performed Kolmogorov–Smirnov test (KS-test) for each locus to compare its IPD distribution with the IPD distribution at the same locus in the WGA sample, and used the estimated DNA modification proportion of the locus with the smallest *P*-value reported by KS-test as the DNA modification proportion of the modified locus in the window. The rationale of this approach is that a smaller *P*-value indicates that the locus has larger effect size *d*_*k*_, which leads to higher estimation accuracy (see the results on simulated data sets). The window we used in this study is 10 bases. In case there are two or more modified loci in the same window, DNA modification proportion cannot be estimated accurately because they may affect IPD profile of each other.

### Performance evaluation with simulated data sets

The number of bases covering each locus, denoted by *m*_*k*_, the number of times each base being sequenced, denoted by *n*_*ki*_, and the effect size, denoted by *d*_*k*_ (see the definition in Equation (5)), are three major factors that affect the performance of DNA modification proportion estimation algorithms. *m*_*k*_ and *n*_*ki*_ can be tuned by users when designing SMRT sequencing experiments. Given coverage of the *k*th locus, $\sum _{i=1}^{m_k}n_{ki}$, larger *n*_*ki*_ leads to smaller *m*_*k*_ and smaller *n*_*ki*_ leads to larger *m*_*k*_. Because of the circular DNA library SMRT sequencing adopted, shorter DNA fragment length leads to more times each base being sequenced (larger *n*_*ki*_) and larger DNA fragment length leads to smaller *n*_*ki*_. *m*_*k*_ and *n*_*ki*_ can be adjusted by choosing DNA fragment length.

**Figure 2.**
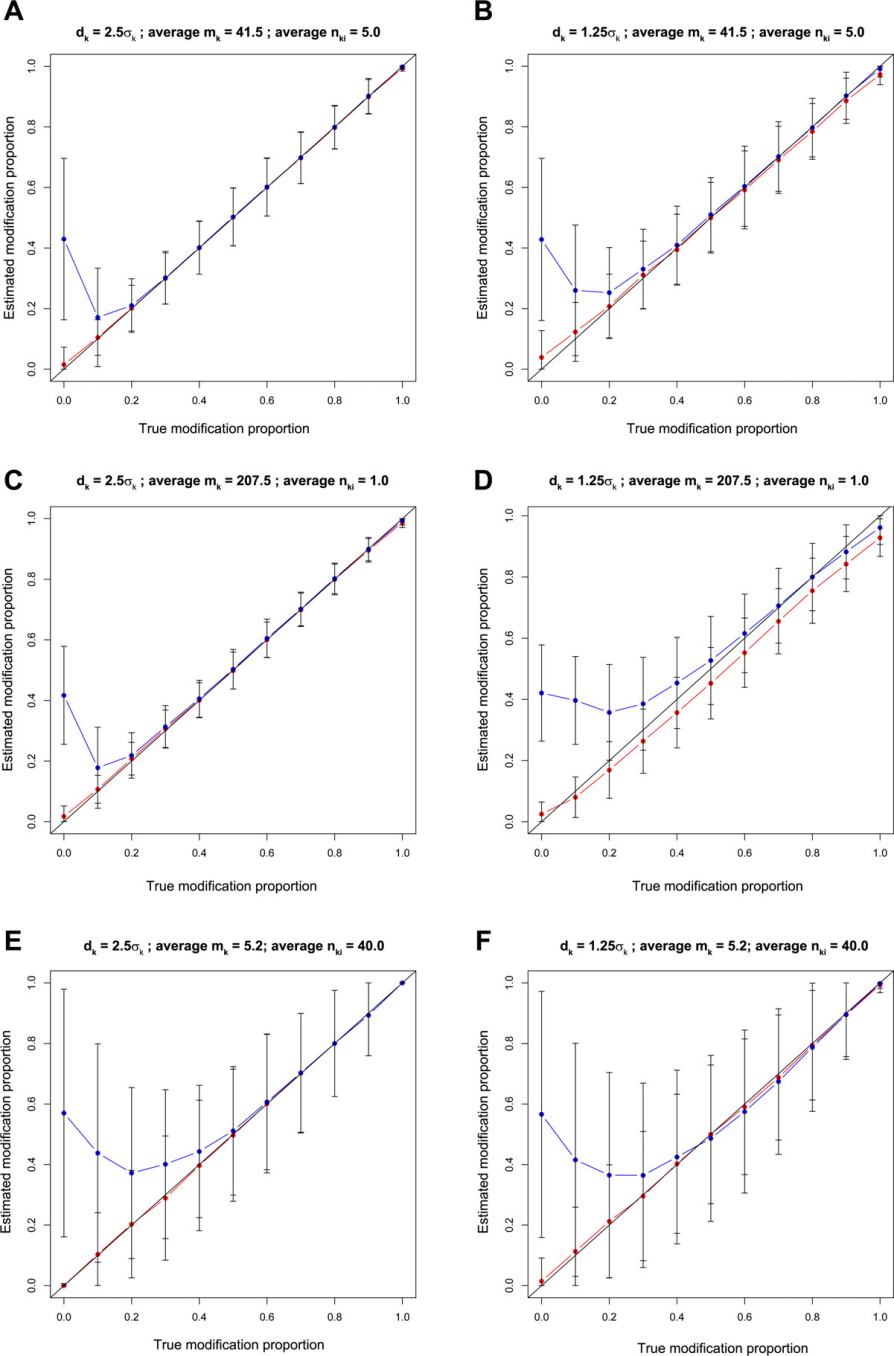
Performance comparison between the empirical Bayes mixture model and the standard mixture model on the simulated datasets. The blue and red curves are the estimated modification proportions of the standard mixture model and the empirical Bayes mixture model, respectively. Each dot is the mean of estimated modification proportion, each upper error bar is mean + standard deviation (is the maximum if mean + standard deviation is larger than the maximum), and each lower error bar is mean − standard deviation (is the minimum if mean − standard deviation is smaller than the minimum). (**A**) The results on the data sets where *d*_*k*_ = 2.5σ_*k*_, average *m*_*k*_ = 41.5 and average *n*_*ki*_ = 5.0. (**B**) The results on the data sets where *d*_*k*_ = 1.25σ_*k*_, average *m*_*k*_ = 41.5 and average *n*_*ki*_ = 5.0. (**C**) The results on the data sets where *d*_*k*_ = 2.5σ_*k*_, average *m*_*k*_ = 207.5 and average *n*_*ki*_ = 1.0. (**D**) The results on the data sets where *d*_*k*_ = 1.25σ_*k*_, average *m*_*k*_ = 207.5 and average *n*_*ki*_ = 1.0. (**E**) The results on the data sets where *d*_*k*_ = 2.5σ_*k*_, average *m*_*k*_ = 5.2 and average *n*_*ki*_ = 40.0. (**F**) The results on the data sets where *d*_*k*_ = 1.25σ_*k*_, average *m*_*k*_ = 5.2 and average *n*_*ki*_ = 40.0.

By fixing coverage, we constructed six simulated data sets (Table 1) to compare the empirical Bayes mixture model with the standard mixture model that does not leverage genome-wide kinetic information (see the Supplemental Methods for details): (i) *d*_*k*_ = 2.5σ_*k*_, average *m*_*k*_ = 41.5 and average *n*_*ki*_ = 5.0 (Figure 2A); (ii) *d*_*k*_ = 1.25σ_*k*_, average *m*_*k*_ = 41.5 and average *n*_*ki*_ = 5.0 (Figure 2B); (iii) *d*_*k*_ = 2.5σ_*k*_, average *m*_*k*_ = 207.5 and average *n*_*ki*_ = 1.0 (Figure 2C); (iv) *d*_*k*_ = 1.25σ_*k*_, average *m*_*k*_ = 207.5 and average *n*_*ki*_ = 1.0 (Figure 2D); (v) *d*_*k*_ = 2.5σ_*k*_, average *m*_*k*_ = 5.2 and average *n*_*ki*_ = 40.0 (Figure 2E); and (vi) *d*_*k*_ = 1.25σ_*k*_, average *m*_*k*_ = 5.2 and average *n*_*ki*_ = 40.0 (Figure 2F). Details of simulated data sets construction are given in the Materials and Methods section.

The results in Figure 2 show that the standard mixture model tends to overestimate DNA modification proportion and has large variance when true DNA modification proportion is low. This is because the standard mixture model needs to estimate two parameters *μ*_*k*1_ and *p*_*k*_ only based on data of the *k*th locus $\mathbf {y_k}$, and for loci covered by no kinetic variant base, no information is available to estimate *μ*_*k*1_, which is mean of IPD distribution of kinetic variant bases covering the *k*th locus. Estimated DNA modification proportions of the empirical Bayes model are close to the true values in most of the data sets. However, when effect size is small (*d*_*k*_ = 1.25σ_*k*0_) and each base is sequenced only once, the empirical Bayes model underestimates DNA modification proportion (Figure 2D). This can be remedied by sequencing each base multiple times (Figure 2B and F).

The results on simulated data sets not only demonstrate superior performance of the empirical Bayes mixture model compared with the standard mixture model but also highlight the tradeoff between *m*_*k*_ and *n*_*ki*_ when coverage is fixed. Sequencing each base only once can lead to a good accuracy when the effect size, *d*_*k*_, is large (Figure 2B). Increasing *n*_*ki*_ (*m*_*k*_ decreases because the coverage is fixed) leads to higher variance of qDNAmod's estimation (Figure 2A and E). However, small *n*_*ki*_ leads to biased estimation when effect size, *d*_*k*_, is small (Figure 2D). A modest *n*_*ki*_ is needed to reduce the bias (Figure 2B), but sequencing each base a large number of times leads to high variance of the estimation (Figure 2F).

### Quantitative detection of DNA modification heterogeneity in *S. pneumoniae* ST556

#### Discovering DNA modification motifs

We performed SMRT sequencing analysis with the genomic DNA of *S. pneumoniae* strain ST556. To detect potential growth phase-dependent DNA modifications, we extracted genomic DNA from *S. pneumoniae* ST556 at lag (OD_620_= 0.08), log (OD_620_= 0.5), and stationary (OD_620_=0.8) phases. The native DNA samples were sequenced by SMRT sequencing without DNA amplification. A whole genome amplified (WGA) DNA sample was sequenced as a modification-negative control (Table 2). The reads were mapped to the reference genome of *S. pneumoniae* ST556 (accession CP003357) by BLASR (basic local alignment with successive refinement) (33) and the DNA modification proportion at each locus was calculated by qDNAmod.

**Figure 3.**
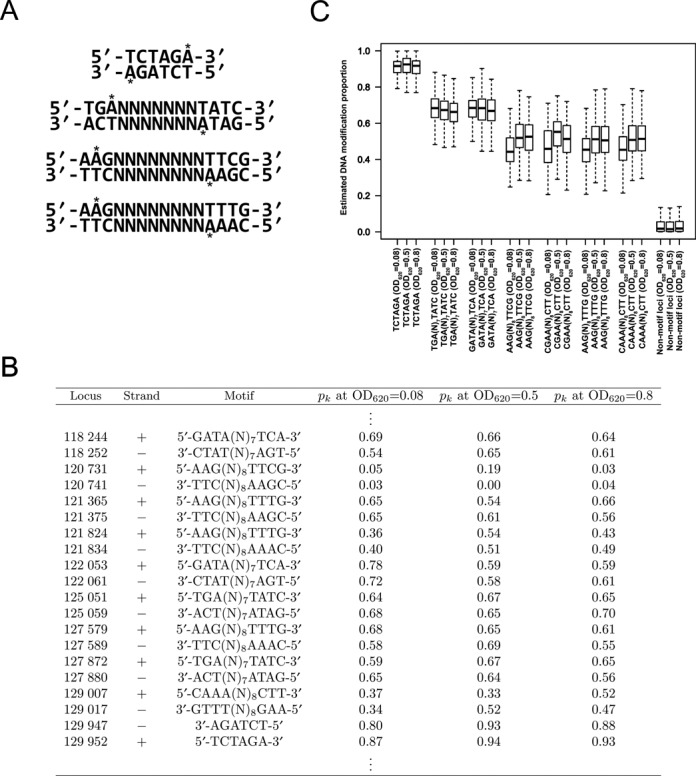
Revelation of intercellular heterogeneity in methylation status of bacterial genome by qDNAmod. (**A**) Novel DNA methylation motifs in the genomes of *S. pneumoniae* ST556 identified by the SMRT sequencing analyses. The methylated bases are marked with asterisks. (**B**) Proportions of methylated adenine residues in the target loci of the motifs between nucleotides 118 244 and 129 952 of the ST556 genome (accession CP003357) as estimated by qDNAmod. (**C**) Box plots of estimated DNA modification proportions at target loci of the motifs. DNA modification proportion at a locus means proportion of cells whose DNA molecule is modified at the locus. The SMRT sequencing data of *S. pneumoniae* ST556 were processed by qDNAmod to calculate the modification proportion at each genome locus. In each box plot, the box is interquartile range (the upper edge is the third quartile (Q3) and the lower edge is the first quartile (Q1)). The horizontal line in the box is the median. The upper vertical bar is Q3 + 1.5(Q3 − Q1) if it is smaller than the maximum, otherwise is the maximum. The lower vertical bar is Q1 − 1.5(Q3 − Q1) if it is larger than the minimum, otherwise is the minimum.

We regarded the loci as significantly modified when their estimated DNA modification proportions are higher than 0.3. The input of MoSDi (Motif Statistics and Discovery), a pattern-driven motif discovery method (34), consisted of DNA sequences in the ±20 base windows (from 20 bases upstream to 20 bases downstream) of the significantly modified loci. By restraining maximal and minimal motif size to 20 bases and four bases, respectively, we identified four highly significant motifs: 5′-TCTAGA-3′, 5-TGA(N)_7_TATC-3, 5-AAG(N)_8_TTTG-3 and 5-AAG(N)_8_TTCG-3 (N represents any of the four nucleotides) (Figure 3A). According to the IPD profiles of various types of DNA modifications (15), the SMRT sequencing data indicated that the modifications in these motifs contained methylation of adenines (6-mA). The precise locations of these motifs in the genome were further mapped (Supplementary Tables S4–S17). Based on the characteristics of the R-M recognition sequences (35), one of these motifs (5′-TCTAGA-3′) is identical to the recognition sequence of XbaI, a type II R-M restriction enzyme, while the others resembled those of type I R-M systems.

#### Validation of methyltransferase recognition motifs

**Figure 4.**
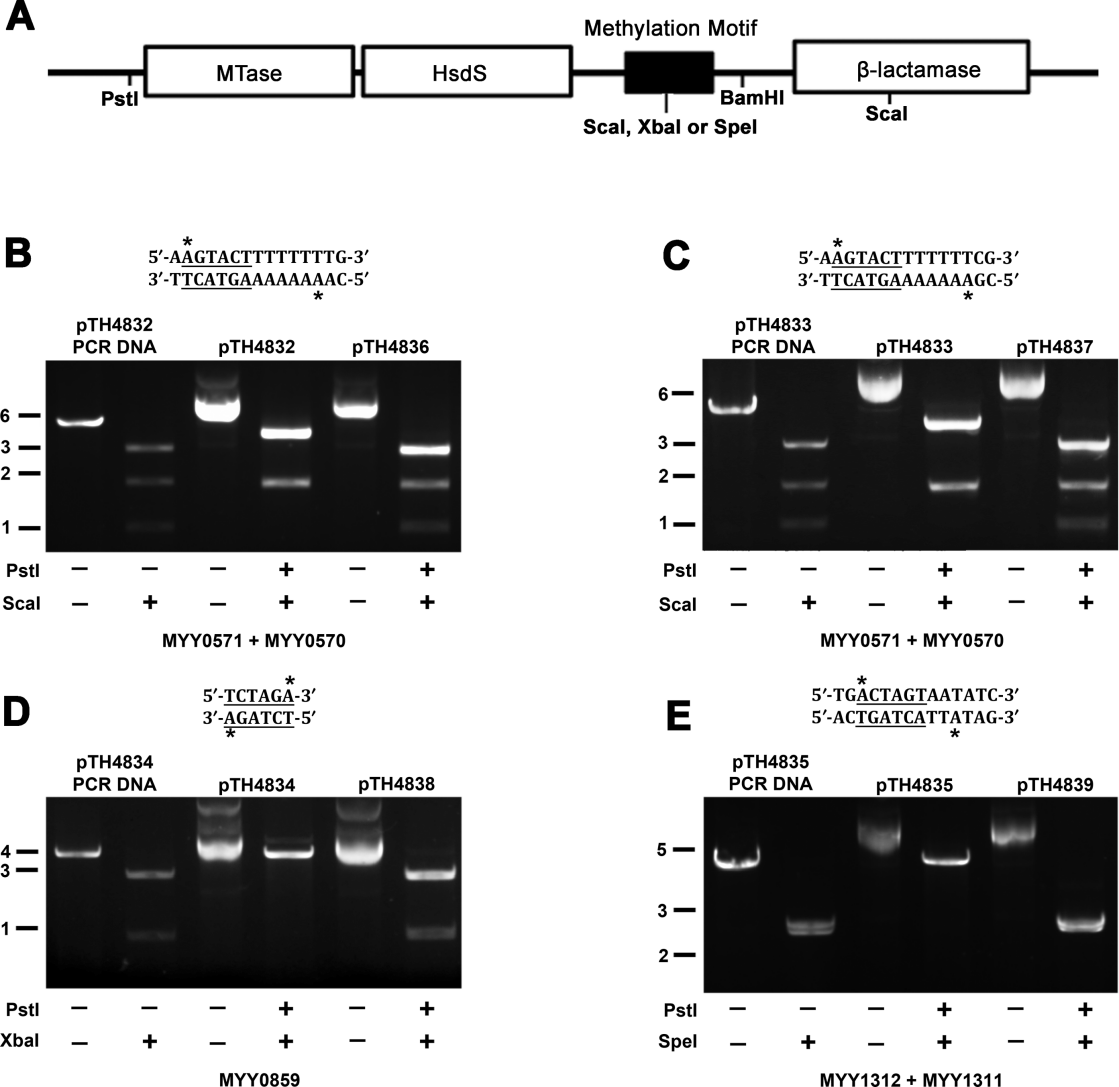
Detection of the recognition sites of the cloned methyltransferase genes from *S. pneumoniae* ST556 by a methylation protection assay. (**A**) A diagrammatic illustration of the strategy for cloning type I R-M *hsdM* and adjacent *hsdS* genes or type II MTase gene, and methylation motifs (detected by SMRT sequencing) into the PstI-BamHI sites of pRRS. (**B**) Plasmid pTH4832 containing *hsdM* (MYY0571), *hsdS* (MYY0570) and methylation motif 5′-**A****AG**TACTTTTT**TTTG**-3′ was isolated from *E. coli* ER2796, treated in the presence (+) or absence (−) of restriction enzymes, and separated by agarose gel electrophoresis. PCR-amplified pTH4832 and plasmid pTH4836 containing the frame-shifted *hsdM* (MYY0571) were included as the controls for unmethylated DNA and inactive MTase, respectively. Methylated adenine residue and recognition sequence for ScaI are marked by asterisks and underlining, respectively. (**C**) Similar to (B), except that the methylation motif 5′-**A****AG**TACTTTTT**TTTG**-3′ was replaced with another sequence 5′-**A****AG**TACTTTTT**TTCG**-3′. (**D**) Similar to (B), except for using a separate set of type I R-M *hsdM* (MYY1312)-*hsdS* (MYY1311) and a cognate methylation motif 5′-**TG****A**CTAGTAA**TATC**-3′ for cloning in pRRS (resulting in pTH4834). PCR-amplified pTH4835 and plasmid pTH4839 containing the frame-shifted MYY1312 were used as the controls for unmethylated DNA and inactive HsdM, respectively. (**E**) Similar to (B), except for using pTH4834 containing the type II R-M MTase MYY0859 and a cognate methylation motif 5′-TCTAGA-3′. PCR-amplified pTH4834 and plasmid pTH4838 containing the frame-shifted MYY0859 were digested as the controls for unmethylated DNA and inactive MTase, respectively. Molecular sizes of the standards are indicated in kilobases (kb).

To identify the methyltransferases that are responsible for methylating the motifs in the *S. pneumoniae* ST556 genome detected by SMRT sequencing (Figure 3A), we tested the activities of putative type I and II R-M systems in *S. pneumoniae* ST556, in terms of methylating these motifs. Based on the predicted R-M systems in the REBASE database (http://tools.neb.com/∼vincze/genomes/report.php?genome_id=13214) (Figure 4A), we cloned the genes encoding three putative DNA methyltransferases in the predicted type I (MYY0571 and MYY1312) and II (MYY0859) R-M systems in the plasmid pRRS, a vector that was previously used to study DNA methyltransferase specificity (26). For the predicted type I R-M systems, the host specificity determinant genes (*hsdS*) (MYY0570 and MYY1311) were cloned together with their corresponding host specificity determinant methyltransferases (*hsdM*). Four representative methylation motifs identified by SMRT sequencing (Figure 3A) were inserted in the 3′ ends of the R-M system genes. The recombinant plasmids were isolated from *E. coli* ER2796, a strain lacking all known methyltransferase activities (20), and were tested for the methylation status of the sequence motifs by restriction enzymes that cut unmethylated but not methylated motifs. As shown in Figure 4B, plasmid pTH4832 harboring *hsdM* (MYY0571) and *hsdS* (MYY0570) of a predicted type I R-M system and a methylation motif 5′-**A****AG**TACTTTTT**TTTG**-3′ was cut into two bands by ScaI, indicating this sequence was methylated and protected from the digestion. As a control, the unmethylated PCR products of the plasmid and pTH4836 carrying a frame-shifted *hsdM* (MYY0571) were completely digested into three fragments by ScaI. Similar results were observed with plasmid pTH4833 carrying another methylation motif 5′-**A****AG**TACTTTTT**TTCG**-3′ (Figure 4C). These results indicated that the gene locus of MYY0570-MYY0572 in *S. pneumoniae* ST556 encodes an authentic type I R-M system that recognizes at least two different DNA motifs 5′-AAG(N)_8_TTTG-3′ and 5′-AAG(N)_8_TTCG-3′.

Additional experiments revealed that the HsdM (MYY1312) and HsdS (MYY1311) subunits of the second putative type I R-M system in *S. pneumoniae* ST556 protected DNA motif 5′-**TG****A**CTAGTAA**TATC**-3′ in plasmid pTH4835 from restriction digestion by SpeI (Figure 4E). In contrast, SpeI completely digested the same methylation motif in the unmethylated PCR products of pTH4835 and pTH4839 containing a frame-shifted *hsdM* (MYY1312). We conclude that the MYY1306-MYY1312 gene locus of *S. pneumoniae* ST556 encodes a functional type I R-M system that recognizes the DNA motif 5′-TGA(N)_7_TATC-3′. In a similar trial, the plasmid carrying MYY0859 blocked the digestion of the DNA motif 5′-TCTAGA-3′ by XbaI (Figure 4D), indicating that the protein encoded by MYY0859 represents a functional DNA MTase. This results implies that the MYY0859-MYY0860 gene locus of *S. pneumoniae* ST556 encodes an authentic type II R-M system as predicted in REBASE.

In summary, this study, for the first time, has experimentally identified three novel R-M systems in *S. pneumoniae*. We designated these newly characterized DNA methyltransferases M. Spn556III (MYY0571; type I R-M), M. Spn556IV (MYY0859; type II R-M) and M. Spn556V (MYY1312; type II R-M). The type II R-M system Spn556IV is an isoschizomer of XbaI. These nomenclatures were made after those of DpnI and DpnII, two known type II R-M systems of *S. pneumoniae* (36).

#### Detection of DNA methylation status

We used qDNAmod to assess the DNA methylation proportions for the four R-M recognition motifs identified from the SMRT sequencing data of ST556 (Figure 3A). For a motif, we refer to the locus that can be modified as a target locus of the motif. The modification proportions were estimated for each target locus of the four motifs (modified molecules/total molecules at a single genome locus) by qDNAmod (Figure 3B) and listed in Supplementary Tables S4–S17.

For the convenience of comparison and analysis, we also calculated the distributions of methylation proportion of all target loci for each motif with the sequencing data from the forward and reverse strands. As represented in Figure 3C, the median methylation proportion for the target loci of the type II R-M motif 5′-TCTAGA-3′ is 0.92 for three ST556 clonal samples, suggesting that the vast majority of the loci were fully modified in the pneumococcal cells under three different growth phases. The median methylation proportions of the three type I R-M motifs are: 5-TGA(N)_7_TATC-3 (median *p*_*k*_ = 0.62), 5-AAG(N)_8_TTCG-3 (median *p*_*k*_ = 0.50) and 5-AAG(N)_8_TTTG-3 (median *p*_*k*_ = 0.49). Statistical analyses showed that these type I R-M motifs were methylated at significantly lower proportions than the type II motif. (We performed Welch's *t*-test to compare the methylation proportions of the three type I R-M motifs with the methylation proportions of the type II R-M motifs and obtained *P*-values less than 10^−16^ for each of the type I R-M motifs.) The total number of occurrences of the partially modified motifs in the genome is 1646. These results demonstrate that intercellular DNA modification heterogeneity is widespread in the *S. pneumoniae* ST556 genome, whose size is 2 145 902 bp.

As shown in Figure 3C, the target loci of the three motifs for the two type I R-M systems are not fully methylated. As SMRT sequencing-based DNA modification detection is strand-specific, there are four types of potential modification statuses that may explain the partial modification result for each given motif: both DNA strands modified, both strands unmodified, only the forward strand is modified and only the backward strand is modified (Supplementary Figure S3). We used a correlation analysis to determine which of four types of modification status exist in *S. pneumoniae* ST556 genome. Assuming that the *k*th locus on the forward strand is the target locus of motif 5′-TGA(N)_7_TATC-3′ and the *k*′ locus on the backward strand is the target locus of complementary part of this motif, 3′-ACT(N)_7_ATAG-5′, the Pearson correlation coefficient (PCC) between the average IPDs of bases covering the *k*th locus and the *k*th locus from the same DNA molecule was calculated (details are given in Supplementary Figure S4). It should be noted that all template DNA molecules in the SMRT sequencing library are circularized (27,28). With these considerations, only the locus where the modification statuses on both stands are the same would yield a positive PCC value, whereas that with modification on only one of the DNA strands would lead to a negative PCC.

By calculating PCCs for all target loci of the three partially modified motifs, we obtained positive and high PCC values for each of the three motifs (Supplementary Figure S5A), thus suggesting that both DNA strands are methylated in the same manner for a given target locus of these motifs. Namely, the target loci with only one of the two strands methylated are rare or do not exist. The examples of the three partially modified motifs are shown in Supplementary Figure S5B–D.

**Figure 5.**
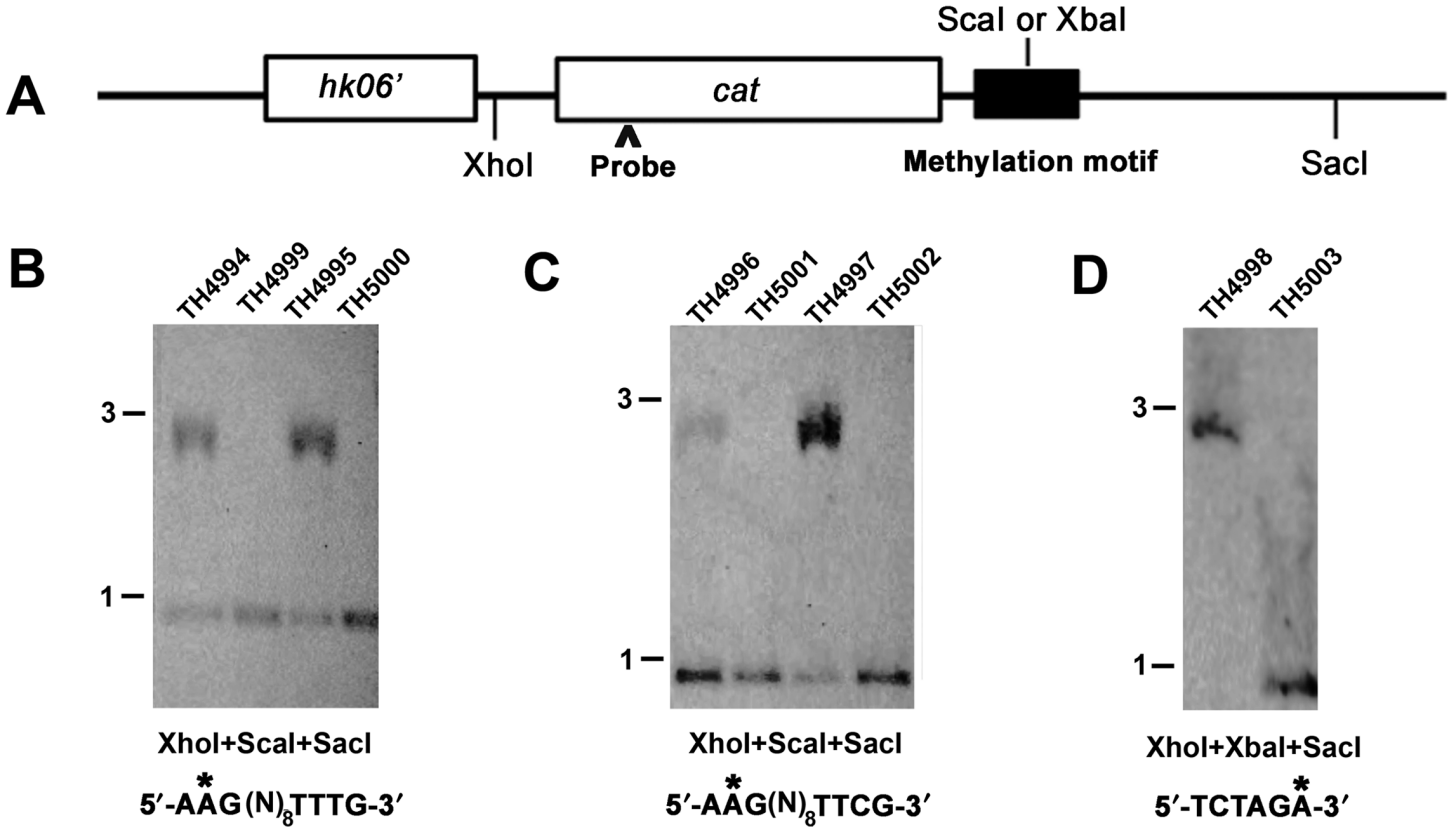
Incomplete methylation of type I R-M system methylation motifs in *S. pneumoniae* ST556 genome as determined by Southern hybridization. (**A**) Illustration of the plasmid pST393 derivatives that carried the representative types I and II R-M system methylation motifs of *S. pneumoniae* ST556. (**B**) Genomic DNA samples from *S. pneumoniae* ST556 derivatives carrying different methylation motifs were treated with XhoI, SacI and methylation-sensitive ScaI or XbaI and separated by agarose gel electrophoresis. The resulting DNA blots were hybridized with the probe from the chloramphenicol acetyltransferase gene (cat) of the plasmid pST393. Methylated residues are marked by asterisks. (**C**) Similar to (B), except for using another methylation motif 5′-AAG(N)_8_TTCG-3′. (**D**) Similar to (B), except for using a type II MTase MYY0859 and its cognate methylation motif 5′-TCTAGA-3′. Molecular sizes of the standards are indicated in kb. The probe is marked by an arrowhead.

We performed Southern blotting analysis to verify the existence of partial DNA methylation on the chromosome of *S. pneumoniae* ST556. For this purpose, two different type I methylation motifs (5′-AAG(N)_8_TTTG-3′ and 5′-AAG(N)_8_TTCG-3′) and one type II methylation motif (5′-TCTAGA-3′) were chosen to detect methylation status by digestion with methylation-sensitive restriction enzymes (e.g. ScaI and XbaI). The results in Figure 5 showed that the unmethylated form of these sequences was effectively cleaved by ScaI (Figure 5B and C) or XbaI (Figure 5D) in a highly specific manner, but the same digestions could be blocked to an undetectable level by the presence of the corresponding DNA methyltransferases. These methylation motifs were cloned into the suicide vector pST393 and inserted into the chromosome of *S. pneumoniae* ST556 or isogenic R-M system-deficient mutants by natural transformation and homology recombination.

As illustrated in Figure 5A, the genomic DNA preparation of the resulting *S. pneumoniae* ST556 derivatives was effectively digested by XhoI, SacI and a methylation-sensitive restriction enzyme (ScaI or XbaI). The probe (plasmid sequence) detected two DNA fragments from *S. pneumoniae* ST556 derivative (TH4994, ST556::pTH4994) with the intact type I R-M system MYY0570-MYY0572 (Figure 5B). In contrast, the isogenic strain (TH4999) with a deletion in the type I R-M system MYY0570-MYY0572 showed only an ∼1-kilobase (kb) band (XhoI-ScaI fragment, 0.904 kb) without any detectable signal for the ∼3-kb band (the XhoI-SacI fragment, 2.889 kb). This result indicated that the DNA motif (5′-**A****AG**TACTTCGG**TTTG**-3′) is incompletely methylated and thereby partially digestible by ScaI in the presence of the type I R-M system MYY0570-MYY0572 (strain TH4994) and that knocking out the type I R-M system led to the loss of methylation in the same sequence (strain TH4999). We verified this result with another sequence (5′-**A****AG**TACTTTTT**TTTG**-3′) of the same methylation motif (5′-AAG(N)_8_TTTG-3′). The strains with (TH4995) and without (TH5000) the type I R-M system showed the same restriction digestion patterns (Figure 5B, right half). We also tested the digestibility of two representative sequences (5′-**A****AG**TACTTGAG**TTCG**-3′ and 5′-**A****AG**TACTTTTT**TTCG**-3′) of the methylation motif (5′-AAG(N)_8_TTCG-3′) for the same type I R-M system. Similar results were obtained with the *S. pneumoniae* ST556 derivatives in the presence (TH4996 and TH4997) or absence (TH5001 and TH5002) of the type I R-M system.

Since nearly complete methylation was detected for all methylation motifs of the type II R-M system MYY0859-MYY0860, we performed similar Southern blotting analysis with TH4998 (ST556::pTH4998) and TH5003 (ST556::pTH4998, ΔMYY0859-MYY0860). In the presence of the type II R-M system (TH4998), only one ∼3-kb band was observed (Figure 5D), representing the size of the XhoI-SacI fragment (2.889 kb). This result indicated the complete protection of the methylation motif from XbaI digestion by DNA methylation. Consistently, knocking out the type II R-M system (strain TH5003) led to the complete digestion of the methylation motif by XbaI as reflected by the ∼1-kb band (the XhoI-XbaI fragment, 0.902 kb). Taken together, these observations demonstrated incomplete methylation of the DNA motifs for the type I R-M system MYY0570-MYY0572, thus providing the experimental evidence for quantitative detection of heterogeneity in DNA methylation status from SMRT sequencing data by qDNAmod.

## DISCUSSIONS

Phenotypic heterogeneity among individual cells of isogenic or clonal populations frequently occurs in both prokaryotic and eukaryotic organisms and fulfills many critical biological functions. DNA modification is an important epigenetic factor that contributes to intercellular phenotypic heterogeneity (2,37). It has been demonstrated that SMRT sequencing is capable of detecting various DNA modifications and intercellular heterogeneity of DNA modification at the genome scale. However, the existing methods for detecting DNA modification with SMRT sequencing data only report whether a genome locus is modified at a cell population level but neglect intercellular heterogeneity. Thus, there is an urgent need for bioinformatic tools that can quantitatively detect intercellular heterogeneity of DNA modification from SMRT sequencing data. In this study, the combination of computational and experimental tests showed that qDNAmod is a reliable and effective tool for this purpose.

The results from the simulated data sets showed that the empirical Bayes mixture model employed by qDNAmod has significantly higher accuracy than the standard mixture model in case the true modification proportions are low, and has compatible accuracy with the standard mixture model in case the true modification proportion is medium or high. In case the effect size is small and each base is sequenced few times, the empirical Bayes mixture model tends to underestimate the modification proportion. This can be remedied by sequencing each base more times.

Design of sequencing experiments is critical for reliable detection of DNA modification heterogeneity by SMRT sequencing. The number of bases covering each locus, *m*_*k*_, and the number of times each base being sequenced, *n*_*ki*_, are important user-defined parameters affecting accuracy of DNA modification proportion detection (Figure 2). According to the principle of SMRT sequencing (27,28), shorter DNA fragment length leads to smaller *m*_*k*_ and larger *n*_*ki*_, but longer DNA fragment length leads to the opposite. We quantitatively studied the trade-off between *m*_*k*_ and *n*_*ki*_ given a fixed sequencing throughput (the total number of sequenced bases) and provide a guideline for choosing *m*_*k*_ and *n*_*ki*_.

qDNAmod is capable of detecting intercellular heterogeneity of DNA methylation (6-mA) from the SMRT sequencing data of bacterial clonal populations. The qDNAmod results predicted that there is ubiquitous intercellular heterogeneity of genome methylation (6-mA) in the *S. pneumoniae* ST556 genome in a clonal population, which implies the existence of multiple subpopulations. The qDNAmod analysis thus indicates the existence of multiple subpopulations within clonal pneumococcal populations in terms of genome modification. The further analysis of the SMRT sequencing results led to the discovery of multiple DNA motifs with 6-mA nucleotides.

Our observations on global partial methylation imply that the corresponding R-M system is inactive in the cells with the unmethylated target motifs. There are three components in a typical type I R-M system (including the type I R-M systems in this study): the R gene encoding the restriction enzyme cutting the target motif, the M gene encoding the methyltransferase to methylate the target motif and protect it from being cut and the S gene encoding the DNA recognition protein. Both of the proteins encoded by the R gene and the M gene need to form a complex with the protein encoded by the S gene to be functional (38). In the cells whose motifs are not methylated, the S gene or the whole R-M system might be inactive or cannot function normally. This can be caused by intercellular heterogeneity of DNA sequences of these genes (13).

The qDNAmod predictions are biologically reproducible. We performed restriction enzyme digestion and Southern blotting and identified three novel R-M systems in *S. pneumoniae* ST556 which are responsible for methylating the DNA motifs identified by qDNAmod. The biological experimentation also verified the incomplete methylation for two of the type I R-M recognition motifs predicted by qDNAmod. To the best of our knowledge, qDNAmod is the first statistical model-based tool for detecting intercellular heterogeneity of DNA modification from SMRT sequencing data, and the result reported by qDNAmod on the *S. pneumoniae* ST556 data is the first genome-wide profile of intercellular adenine methylation heterogeneity in bacteria. Our results provide a powerful tool for studying intercellular phenotypic heterogeneity from genome-wide DNA modification.

In this study, we focus on detecting heterogeneity of DNA modification in haploid cells from pooled SMRT sequencing data (sequenced DNA molecules are extracted from a cell pool rather than a single cell). In this case, we can determine that heterogeneity of DNA modification at a locus is caused by intercellular heterogeneity. It should be noted that, in diploid cells or polyploid cells, intercellular heterogeneity cannot be distinguished from intracellular heterogeneity except for some special cases. For example, if two alleles at a locus are heterozygous in diploid cells, intercellular heterogeneity can be distinguished from intracellular heterogeneity for this locus by sequence differences between these two alleles.

Although we designed the empirical Bayes mixture model for analyzing data of SMRT sequencing technology developed by the Pacific Biosciences in this study, the model has the potential to be extended for analyzing data of other single-molecule sequencing technologies. For example, nanopore sequencing has been proven in principle to be able to detect DNA modification directly from raw sequencing data without preprocessing DNA samples (39–41). As nanopore sequencing also uses analog signals for DNA modification detection at single molecular level, the empirical Bayes mixture model we developed has the potential to be applicable to nanopore sequencing data with some extensions.

This study not only presented a bioinformatic tool, qDNAmod, but also built a statistical framework for DNA modification heterogeneity detection from SMRT sequencing data. As SMRT sequencing has the capability for various types of modifications that are unable to be detected in a genome-wide way by traditional methods (for example, 6-mA, 4-mC and DNA damage), our study on quantitative detection of DNA modification heterogeneity opens a door for this unexplored area.

## CONCLUSIONS

In this study, we designed an empirical Bayes mixture model for detecting intercellular heterogeneity of DNA modification from SMRT sequencing data and implemented a bioinformatic tool, qDNAmod, based on this model for the first time. Performance of qDNAmod was tested with simulated data and SMRT sequencing data of *S. pneumoniae* strain ST556. The analyses of the SMRT sequencing data revealed extensive intercellular heterogeneity in methylation status of *S. pneumoniae* genome mediated by the type I R-M systems.

This study thus presents the first statistical model-based bioinformatic tool for quantitative detection of intercellular heterogeneity in genome DNA modifications from SMRT sequencing data.

## DATA ACCESS

The raw SMRT sequencing data listed in Table 2 are available at http://www.ncbi.nlm.nih.gov/sra, under accession number SRX735344, SRX735345, SRX735346 and SRX735347.

## AVAILABILITY

The source code of qDNAmod is available at https://github.com/zhixingfeng/qDNAmod/releases.

## SUPPLEMENTARY DATA

Supplementary Data are available at NAR Online.

SUPPLEMENTARY DATA
