# qDNAmod: a statistical model-based tool to reveal intercellular heterogeneity of DNA modification from SMRT sequencing data

**DOI:** 10.1093/nar/gkv067

**Published:** 2015-01-29

**Authors:** Z. Feng, J. Li, J.R. Zhang, X. Zhang

Nucleic Acids Res. 2014 Dec 16;42(22):13488–99. doi: 10.1093/nar/gku1097

In this article, the authors have incorrectly designated three DNA methyltransferases in *Streptococcus pneumoniae* strain ST556, as shown in Table 1. The correct nomenclatures should be:

- M. Spn556I (MYY0859; type II R-M system recognizing TCTAGA)
- M. Spn556II (MYY0571; type I R-M system recognizing AAG(N)8TTYG)
- M. Spn556III (MYY1312; type I R-M system recognizing TGA(N)7TATC).

**Table 1.** Changes in nomenclature.

Old name*	New name*
M. Spn556**IV**	M. Spn556**I**
M. Spn556**III**	M. Spn556**II**
M. Spn556**V**	M. Spn556**III**

*Changes are shown in bold.

In addition, the recognition sequence of the type I system encoded by MYY570-MMY572 in Figure 3A should be AAG(N)8TTYG.

