# Supplementary material for: qDNAmod: a statistical model-based tool to reveal intercellular heterogeneity of DNA modification from SMRT sequencing data
Source: Nucleic Acids Res. 2014 Nov 17;42(22):13488–99. doi: 10.1093/nar/gku1097 (PMC4267614; doi:10.1093/nar/gku1097)
Supplement: SUPPLEMENTARY DATA [file supp_42_22_13488__index.html]

qDNAmod: a statistical model-based tool to reveal intercellular heterogeneity of DNA modification from SMRT sequencing data — qDNAmod: a statistical model-based tool to reveal intercellular heterogeneity of DNA modification from SMRT sequencing data — SUPPLEMENTARY DATA 

# qDNAmod: a statistical model-based tool to reveal intercellular heterogeneity of DNA modification from SMRT sequencing data

## SUPPLEMENTARY DATA

**Files in this Data Supplement:**

- SUPPLEMENTARY DATA
