# Supplementary material for: qDNAmod: a statistical model-based tool to reveal intercellular heterogeneity of DNA modification from SMRT sequencing data
Source: Nucleic Acids Res. 2014 Nov 17;42(22):13488–99. doi: 10.1093/nar/gku1097 (PMC4267614; doi:10.1093/nar/gku1097)
Supplement: SUPPLEMENTARY DATA [file supp_gku1097_nar-02867-z-2014-File005.zip › Supplementary_Data/supplemental_figureSx.pdf]

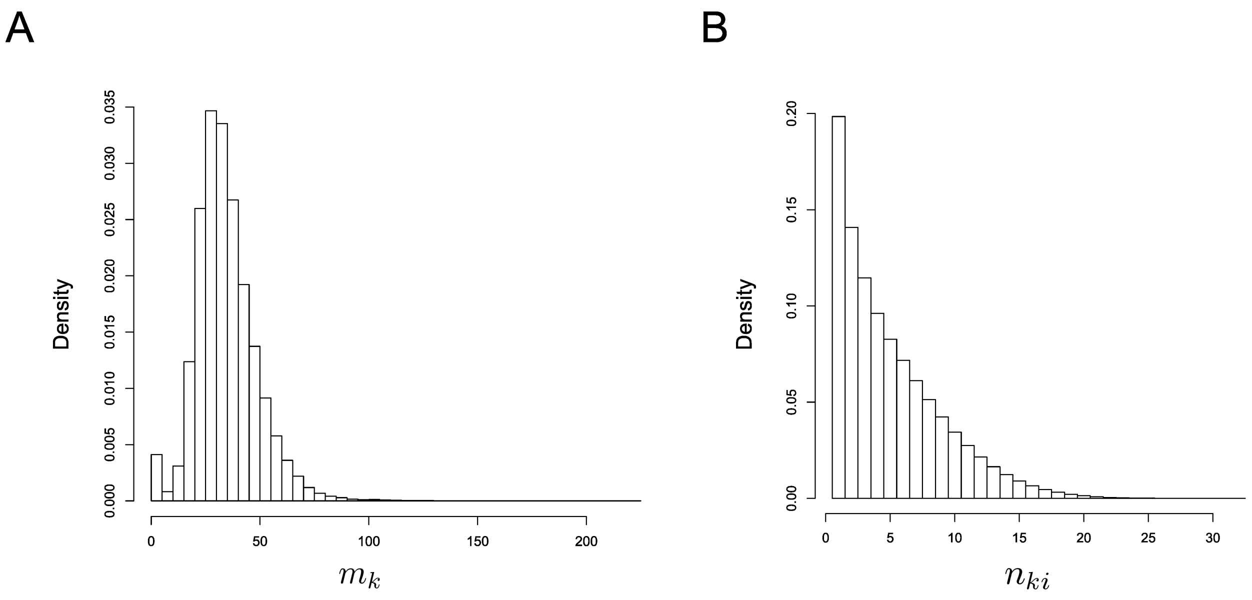

Figure S1: **Histograms of number of bases covering a locus and number of times a base being sequenced estimated from real data**.(A) Histogram of number of bases covering a locus. (B) Histogram of number of times a base being sequenced.

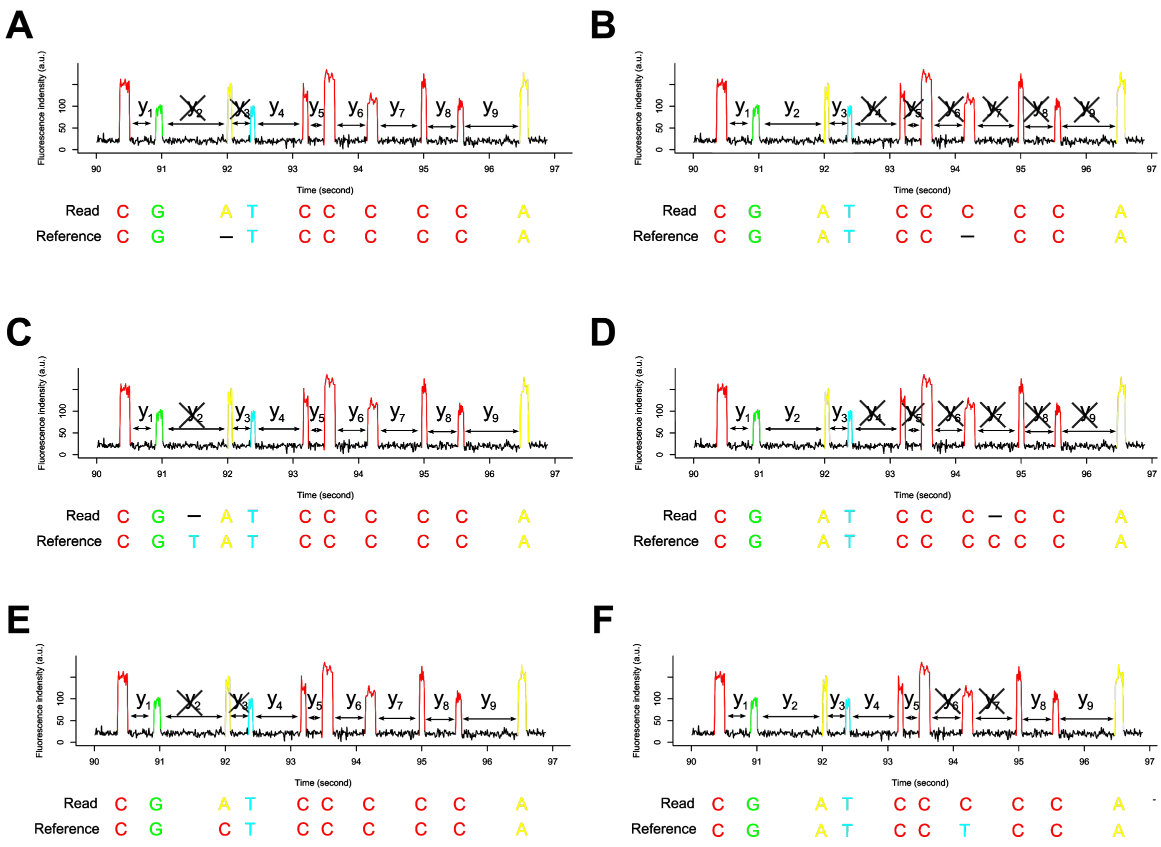

Figure S2: **Data pre-processing**. The "Read" here means the "Incorporating bases" in Figure 1 of the main text. $y_1 \sim y_9$ denote the IPDs. (A) The IPDs of the inserted base and its first downstream base are discarded if the insertion is not in a homopolymer region. (B) The IPDs of all the bases in the homopolymer region are discarded if the insertion is in a homopolymer region. (C) The IPDs of the first downstream base of the deletion are discarded. (D) The IPDs of all the bases in the homopolymer region are discarded if the deletion is in a homopolymer region. (E) The IPDs of the mismatched base and its first downstream base are discarded. (F) Only the IPDs of the mismatched base and its first downstream base are discarded if the mismatch is a homopolymer region.

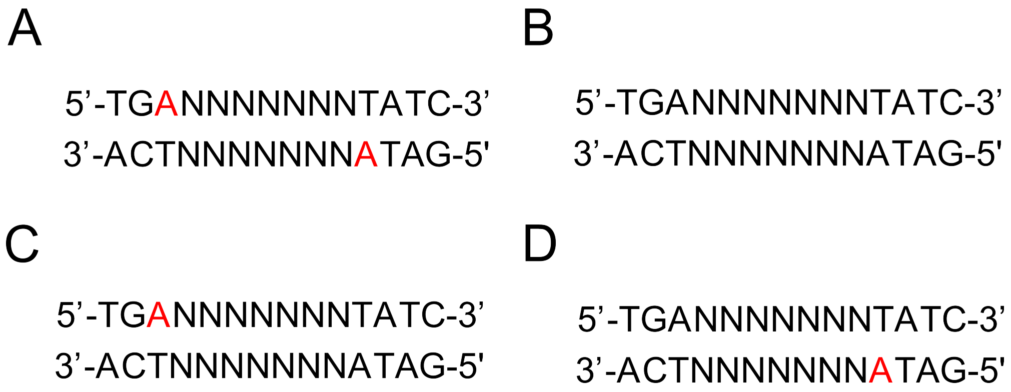

Figure S3: **Four possible DNA modification statuses of a motif**. The red bases are modified bases. (A) Both strands are modified. (B) Both strands are not modified. (C) Only forward strand is modified. (D) Only backward strand is modified.

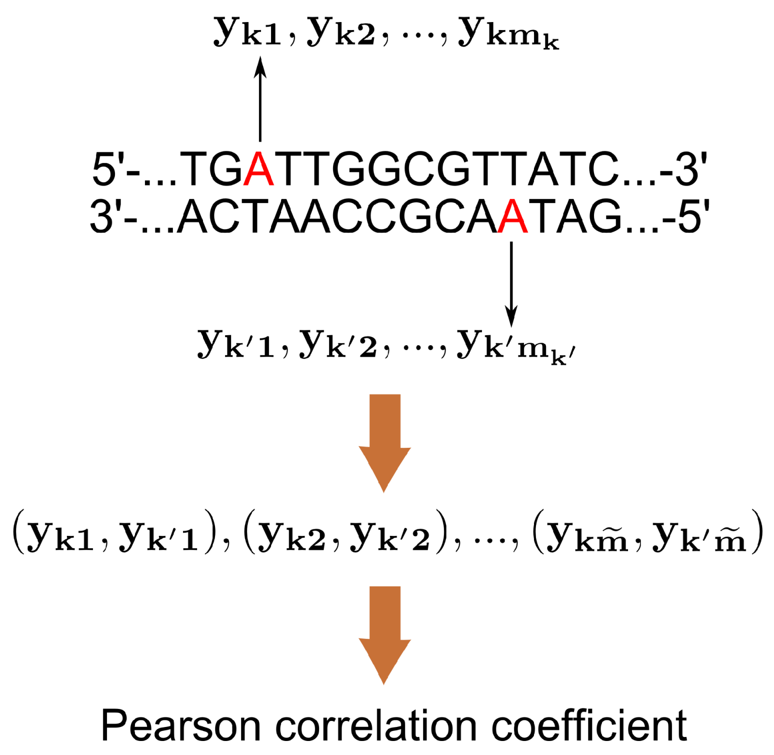

Figure S4: **Correlation analysis to determine mathylation status of a motif**. Assuming that there are m_k bases covering the kth locus whose IPDs are denoted as $\mathbf{y_k} = (\mathbf{y_{k1}}, \mathbf{y_{k2}}, ..., \mathbf{y_{km_k}})$ and its counterpart at the k' locus is covered by $m_{k'}$ bases, whose IPDs are denoted by $\mathbf{y'_k} = (\mathbf{y_{k'1}}, \mathbf{y_{k'2}}, ..., \mathbf{y_{k'm'_k}})$. Without lost of generality, we assume that bases corresponding to each element pair from the first $\tilde{m}$ elements of $\mathbf{y_k}$ and $\mathbf{y_{k'}}$, i.e. $(\mathbf{y_{k1}}, \mathbf{y_{k'1}})$, ..., or $(\mathbf{y_{k\tilde{m}}}, \mathbf{y_{k'\tilde{m}}})$ are from the same molecule. Thus, Pearson correlation coefficient between vectors $(\bar{y}_{k1}, ..., \bar{y}_{k\tilde{m}})$ and $(\bar{y}_{k'1}, ..., \bar{y}_{k'\tilde{m}})$ indicates correlation of modification status between two strand of each molecule, where $\bar{y}_{ki} = \frac{1}{n_{ki}} \sum_{j=1}^{n_{ki}} y_{kij}$, $\bar{y}_{k'i} = \frac{1}{n_{k'i}} \sum_{j=1}^{n_{k'i}} y_{k'ij}$, $i = 1, ..., \tilde{m}$, n_{ki} and $n_{k'i}$ are number of times the ith base covering the kth or k'th locus being sequenced.

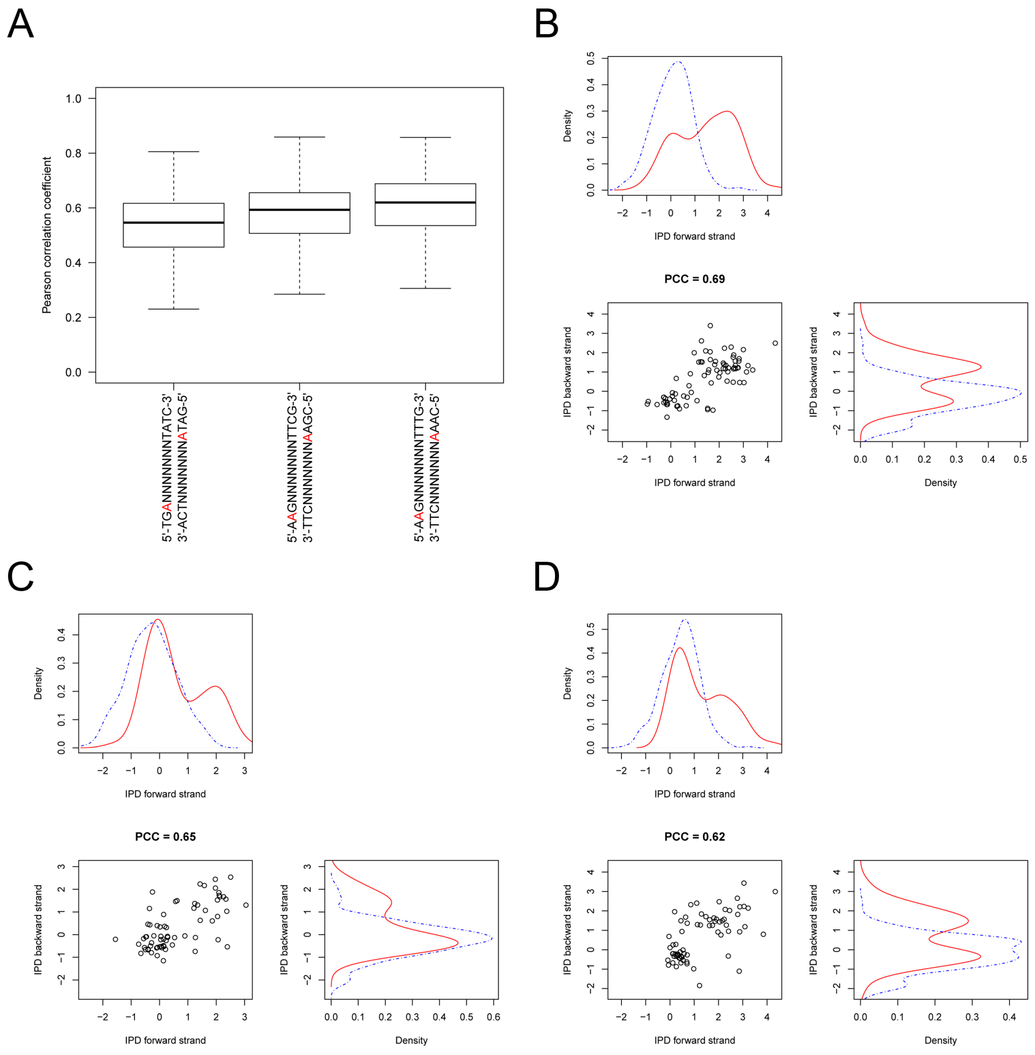

Figure S5: **Heterogeneity of DNA methylation status.** (A) Box plots of Pearson correlation coefficient between IPDs of two complementary target loci of a motif (the red bases of the motifs). (B) The bottom left panel shows the scatter plot of average IPDs of the 28556th locus on the forward stand and the 28564th locus on the backward strand (they are complementary target bases of the same motif) in motif 5'-TGANNNNNNNNTATC-3' (3'-ACTNNNNNNNNATAG-5'). Each dot corresponds to average IPDs of two complementary target bases of a motif in the same molecule. The red curves in the upper left and bottom right panels are estimated IPD distributions of the target bases on the forward strand and backward strand respectively. The blue dashed curves are estimated IPD distribution in the control sample. (C) Similar to (B), The bottom left panel shows the scatter plot of average IPDs of the 48930th locus on the forward stand and 48940th locus on the backward strand in motif 5'-AAGNNNNNNNNNTTCG-3' (3'-TTCNNNNNNNNNAAGC-5'). (D) Similar to (B) The bottom left panel shows the scatter plot of average IPDs of the 2926th locus on the forward stand and the 2936th locus on the backward strand in motif 5'-AAGNNNNNNNNNTTTG-3' (3'-TTCNNNNNNNNNAAAC-5').

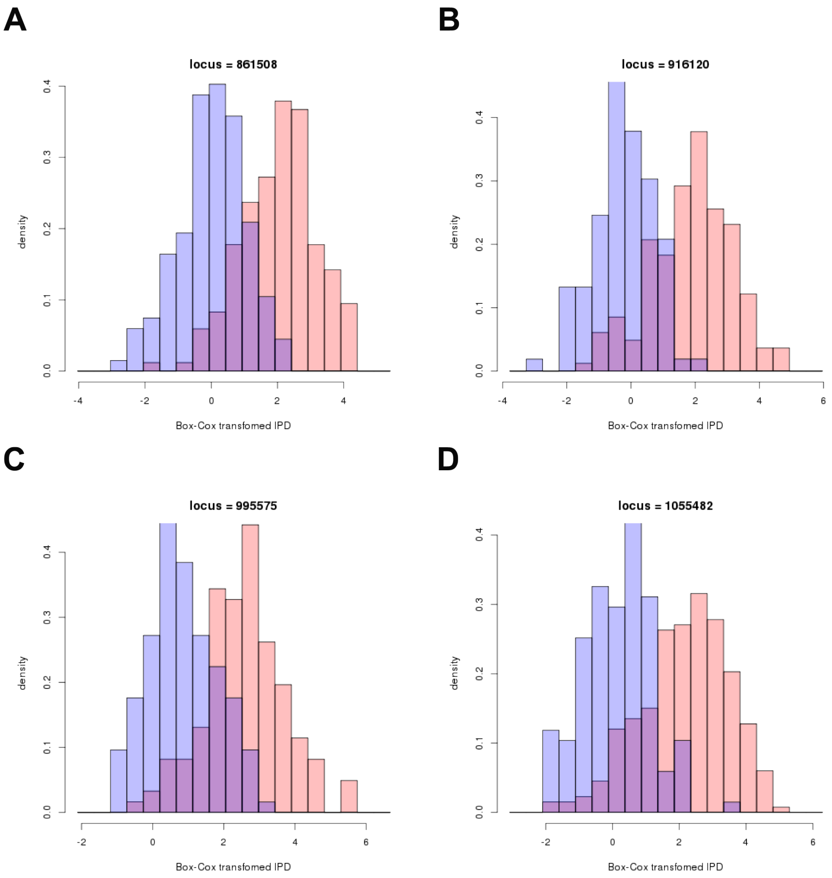

Figure S6: **Impact of DNA modification on Box-Cox transformed IPD.** (A)-(D) show Box-Cox transformed IPD distributions at 4 target loci of motif 5'-TCTAGA-3'. The blue histograms were estimated from the WGA sample and the red ones were estimated from the native sample (OD_{620}=0.08).
