# Supplementary material for: qDNAmod: a statistical model-based tool to reveal intercellular heterogeneity of DNA modification from SMRT sequencing data
Source: Nucleic Acids Res. 2014 Nov 17;42(22):13488–99. doi: 10.1093/nar/gku1097 (PMC4267614; doi:10.1093/nar/gku1097)
Supplement: SUPPLEMENTARY DATA [file supp_gku1097_nar-02867-z-2014-File005.zip › Supplementary_Data/supplementary_methods.pdf]

qDNAmod: a statistical model based tool to reveal intercellular heterogeneity of DNA modification from SMRT sequencing data

Zhixing Feng[1,2†], Jing Li[2†], Jingren Zhang[2*], and Xuegong Zhang[1*]

[1]MOE Key Lab of Bioinformatics/Bioinformatics Division, TNLIST and Department of Automation, Tsinghua University, Beijing 100084, China
[2]Center for Infectious Disease Research, School of Medicine, Tsinghua University, Beijing 100084, China

[*]Correspondence: zhanglab@tsinghua.edu.cn, zhangxg@tsinghua.edu.cn
[†]Equal contributor

Estimating prior distribution of μ_{k1}

The intuitive idea for estimating $g(d_k)$ is to estimate it from IPDs of bases that are likely to be kinetic variant. Formally, we adopted local false discovery rate (local FDR) method introduced in microarray data analysis to estimate $g(d_k)$ [1, 2]. As shown in Figure 1 in the main text (also shown here as Figure 1), for the ith base covering the kth locus, we compared its IPDs with IPDs of the kth locus in the WGA

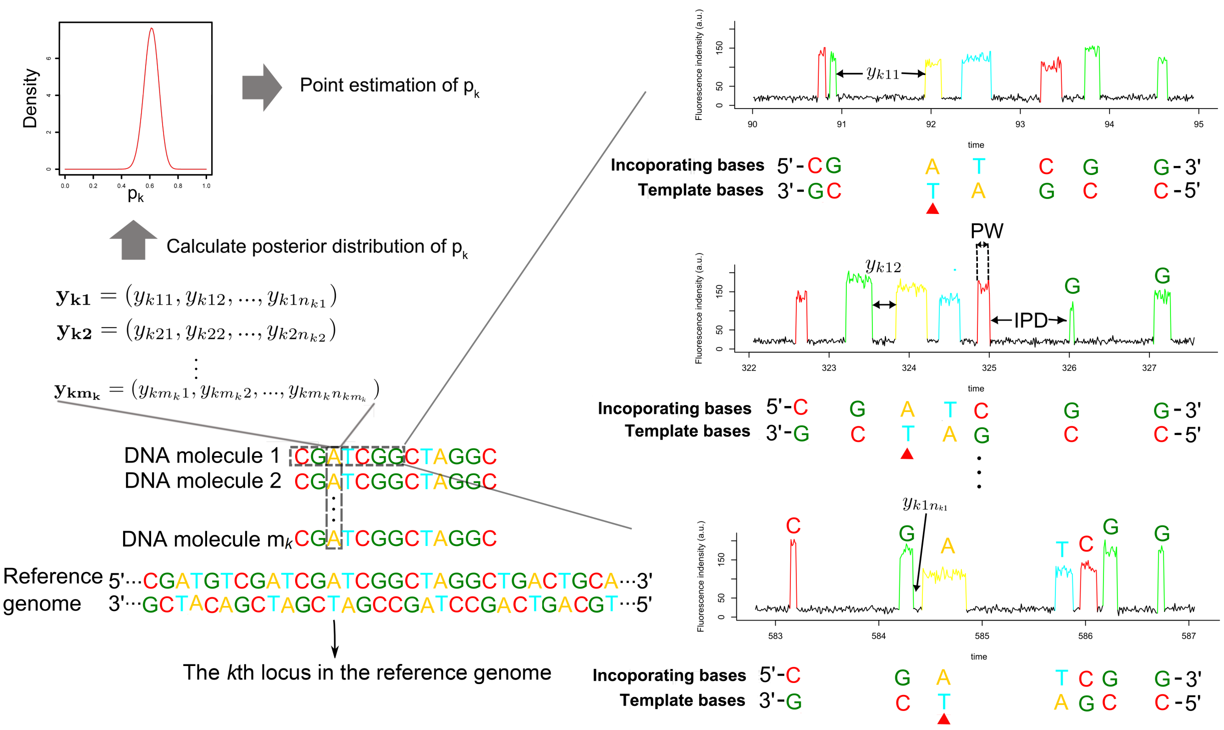

Figure 1: **Quantitative detection of DNA modification heterogeneity.** There are m_k bases covering the kth locus, and they are sequenced n_{k1}, n_{k2}, ..., and n_{km_k} times, respectively. The "T" at the kth locus in the reference genome has a IPD vector, denoted by $\mathbf{y_{k1}}$, $\mathbf{y_{k2}}$,..., $\mathbf{y_{km_k}}$. Given the IPD vector, the posterior distribution of proportion of kinetic variant bases covering the kth locus, p_k, can be estimated by qDNAmod. A point estimation of p_k can be inferred from its posterior distribution.

data and got the student t-statistic defined as

$$t_{ki} = \frac{\bar{y}_{ki} - \bar{y}_k^c}{S_{12}\sqrt{\frac{1}{n_{ki}} + \frac{1}{\sum_{i=1}^{m_k^c} n_{ki}^c}}}$$

where $\mathbf{y^c_{ki}} = (y^c_{ki1}, y^c_{ki2}, ..., y^c_{kin^c_{ki}})$ is the IPDs of the ith base covering the kth locus in the WGA data, and

$$\bar{y}_{ki} = \frac{1}{n_{ki}} \sum_{j=1}^{n_{ki}} y_{kij} \quad (1)$$

$$\bar{y}^c_k = \frac{1}{\sum_{i=1}^{m^c_k} n^c_{ki}} \sum_{i=1}^{m^c_k} \sum_{j=1}^{n^c_{ki}} y^c_{kij} \quad (2)$$

$$S_{12} = \sqrt{\frac{(n_{ki}-1)S_1^2 + (\sum_{i=1}^{m^c_k} n^c_{ki} - 1)S_2^2}{n_{ki} + \sum_{i=1}^{m^c_k} n^c_{ki} - 2}}$$

$$S_1^2 = \frac{1}{n_{ki}-1} \sum_{j=1}^{n_{ki}} (y_{kij} - \bar{y}_{ki})^2$$

$$S_2^2 = \frac{1}{\sum_{i=1}^{m^c_k} n^c_{ki} - 1} \sum_{i=1}^{m^c_k} \sum_{j=1}^{n^c_{ki}} (y^c_{kij} - \bar{y}^c_k)^2$$

Degree of freedom of t_{ki} is $df = n_{ki} + \sum_{i=1}^{m^c_k} n^c_{ki} - 2$. By quantile transformation, we got the z-score

$$z_{ki} = \Phi^{-1}(F_{t_{df}}(t_{ki})) \quad (3)$$

where Φ^{-1} is the inverse function of cumulative density function of standard normal distribution, $F_{t_{df}}$ is the cumulative density function of t distribution with degree of freedom df. z_{ki} follows a mixture distribution, whose probability density function is

$$f(z_{ki}) = (1-\pi)f_0(z_{ki}) + \pi f_1(z_{ki})$$

where f_0 is probability density function of z_{ki} when the base is normal, f_1 is probability density function of z_{ki} when the base is kinetic variant and π is genome-wide proportion of kinetic variant bases. $f_0(z_{ki})$ is probability density function of standard normal distribution and $f(z_{ki})$ was estimated by fitting histogram of $\{z_{ki}|k = 1, 2..., N, i = 1, 2, ..., m_k\}$. Assuming most bases are normal, π was estimated by

$$\hat{\pi} \quad = \quad 1 - \frac{\int_{-x_0}^{x_0} \hat{f}(z_{ki}) dz_{ki}}{\int_{-x_0}^{x_0} f_0(z_{ki}) dz_{ki}}$$

where $\hat{f}(z_{ki})$ is estimated $f(z_{ki})$ and $\pi \int_{-x_0}^{x_0} f_1(z_{ki}) dz_{ki} \approx 0$ when $z_{ki} \in [-x_0, x_0]$. We set $x_0 = 0.5$ in the work. We got probability of a base being kinetic variant given z_{ki} based on genome-wide data, which is

$$\hat{Pr}(I_{ki} = 1|z_{ki}) \quad = \quad 1 - \frac{(1 - \hat{\pi}) f_0(z_{ki})}{\hat{f}(z_{ki})}$$

where $I_{ki} = 1$ if the ith base covering the kth locus is kinetic variant, otherwise $I_{ki} = 0$. Based on $\hat{Pr}(I_{ki} = 1|z_{ki})$, $g(d_k)$ was estimated by a weighted histogram approach. For the ith base covering the kth locus, its effect size was estimated by $\hat{d}_{ki} = \bar{y}_{ki} - \bar{y}_k^c$ ($\bar{y}_{ki}$ and $\bar{y}_k^c$ are defined by equations (1) and (2)) and $\{\hat{d}_{ki}|k = 1, 2, ..., N; i = 1, 2, ..., m_k\}$ were put into B bins with equal size, $[b_1, b_2), [b_2, b_3), ..., [b_B, b_{B+1}]$, whose midpoints are $mid_1, mid_2, ..., mid_B$. Effective frequency of a given bin $[b_t, b_{t+1})$ was estimated by $freq_t = \sum_{(k,i) \in \{(k,i)|\hat{d}_{ki} \in [b_t, b_{t+1})\}} \hat{Pr}(I_{ki} = 1|z_{ki})$, where $\{(k, i)|\hat{d}_{ki} \in [b_t, b_{t+1})\}$ means the set of bases whose $\hat{d}_{ki}$ is in $[b_t, b_{t+1})$. Effective frequency $freq_t$ is actually the expectation of number of kinetic variant bases whose $\hat{d}_{ki}$ is in $[b_t, b_{t+1})$. By using smoothing spline to fit effective frequencies $\{freq_t|t = 1, 2, ..., B\}$ by midpoints $\{mid_t|t = 1, 2, ..., B\}$, we got estimated $g(d_k)$, denoted by $\hat{g}(d_k)$. By shifting,

we got estimated $f(\mu_{k1})$, i.e. $\hat{f}(\mu_{k1}) = \hat{g}(\mu_{k1} - \hat{\mu}_{k0})$, where $\hat{\mu}_{k0}$ is estimated from the WGA data.

Variational inference for fitting empirical Bayes mixture model

We introduced vector $\mathbf{I_k} = (I_{k1}, I_{k2}, ..., I_{km_k})$ to indicate if a base is kinetic variant. When the ith base covering the kth locus is kinetic variant $I_{ki} = 1$, otherwise $I_{ki} = 0$ and $\{I_{ki} | i = 1, 2, ..., m_k\}$ are independent and identically distributed. Given p_k, μ_{k1} and $\mathbf{I_k}$, likelihood function of $\mathbf{y_k}$ can be reformulated as

$$\begin{aligned} f(\mathbf{y_k}|p_k, \mu_{k1}, \mathbf{I_k}) &= \prod_{i=1}^{m_k} f(\mathbf{y_{ki}}|p_k, \mu_{k1}, I_{ki}) \qquad (4) \\ f(\mathbf{y_{ki}}|p_k, \mu_{k1}, I_{ki}) &= f(\mathbf{y_{ki}}|\mu_{k1}, I_{ki}) \\ &= N(\mathbf{y_{ki}}|\hat{\mu}_{k0}, \hat{\sigma}^2_{k0})^{(1-I_{ki})} N(\mathbf{y_{ki}}|\mu_{k1}, \hat{\sigma}^2_{k0})^{I_{ki}} \qquad (5) \end{aligned}$$

where $\hat{\mu}_{k0}$ and $\hat{\sigma}^2_{k0}$ are estimated from WGA data, and

$$\begin{aligned} N(\mathbf{y_{ki}}|\hat{\mu}_{k0}, \hat{\sigma}^2_{k0}) &= \prod_{j=1}^{n_{ki}} N(y_{kij}|\hat{\mu}_{k0}, \hat{\sigma}^2_{k0}) \\ N(\mathbf{y_{ki}}|\mu_{k1}, \hat{\sigma}^2_{k0}) &= \prod_{j=1}^{n_{ki}} N(y_{kij}|\mu_{k1}, \hat{\sigma}^2_{k0}) \end{aligned}$$

Let prior distribution of $\mathbf{I_k}$ and p_k to be

$$\begin{aligned} f(p_k) &= U(0, 1) \qquad (6) \\ f(I_{ki}|p_k) &= (1 - p_k)^{1-I_{ki}} p_k^{I_{ki}} \qquad (7) \end{aligned}$$

where $U(0,1)$ is probability density function of uniform distribution between 0 and 1.

We use a simpler function

$$q(p_k, \mu_{k1}, \mathbf{I_k}) \quad = \quad q(p_k)q(\mu_{k1})q(\mathbf{I_k}) \tag{8}$$

to approximate posterior distribution $f(p_k, \mu_{k1}, \mathbf{I_k}|\mathbf{y_k})$. To achieve this, we minimize Kullback Leibler divergence between $f(p_k, \mu_{k1}, \mathbf{I_k}|\mathbf{y_k})$ and $q(p_k, \mu_{k1}, \mathbf{I_k})$, i.e. minimize functional

$$\begin{aligned} D(q) &= -\int_{-\infty}^{\infty}\int_{0}^{1}\sum_{\mathbf{I_k}\in\{0,1\}^{m_k}} q(p_k, \mu_{k1}, \mathbf{I_k})\log(\frac{f(p_k, \mu_{k1}, \mathbf{I_k}|\mathbf{y_k})}{q(p_k, \mu_{k1}, \mathbf{I_k})})dp_k d\mu_{k1} \\ &= -\int_{-\infty}^{\infty}\int_{0}^{1}\sum_{\mathbf{I_k}\in\{0,1\}^{m_k}} q(p_k, \mu_{k1}, \mathbf{I_k})\log(\frac{f(p_k, \mu_{k1}, \mathbf{I_k}, \mathbf{y_k})}{q(p_k, \mu_{k1}\mathbf{I_k})})dp_k d\mu_{k1} + \log(f(\mathbf{y_k})) \end{aligned}$$

where $\{0,1\}$ is a set whose elements are 0 and 1, $\{0,1\}^{m_k}$ is Cartesian product of m_k $\{0,1\}$ sets. As $\log(f(y_k))$ and function q are not related, minimizing $D(q)$ is equivalent to maximizing

$$L(q) \quad = \quad \int_{-\infty}^{\infty}\int_{0}^{1}\sum_{\mathbf{I_k}\in\{0,1\}^{m_k}} q(p_k, \mu_{k1}, \mathbf{I_k})\log(\frac{f(p_k, \mu_{k1}, \mathbf{I_k}, \mathbf{y_k})}{q(p_k, \mu_{k1}, \mathbf{I_k})})dp_k d\mu_{k1}$$

Substituting formula (8) into $L(q)$ we can get

$$
\begin{aligned}
L(q) &= \int_{-\infty}^{\infty}\int_{0}^{1}\sum_{\mathbf{I_k}\in\{0,1\}^{m_k}} q(p_k)q(\mu_{k1})q(\mathbf{I_k})\log(f(p_k,\mu_{k1},\mathbf{I_k},\mathbf{y_k}))dp_k d\mu_{k1} \\
&- \int_{0}^{1} q(p_k)\log(q(p_k))dp_k - \sum_{\mathbf{I_k}\in\{0,1\}^{m_k}} q(\mathbf{I_k})\log(q(\mathbf{I_k})) \qquad (9)\\
&- \int_{-\infty}^{\infty} q(\mu_{k1})\log(q(\mu_{k1}))d\mu_{k1} \qquad (10)
\end{aligned}
$$

We adopt a stepwise maximizing strategy to maximize $L(q)$.

Estimating $q(\mathbf{I_k})$ Fixing $q(p_k)$ and $q(\mu_{k1})$ in $L(q)$ (formula (10)), we got

$$
\begin{aligned}
L(q) &= \sum_{\mathbf{I_k}\in\{0,1\}^{m_k}} q(\mathbf{I_k})\log(\widetilde{f}(\mathbf{I_k},\mathbf{y_k})) - \sum_{\mathbf{I_k}\in\{0,1\}^{m_k}} q(\mathbf{I_k})\log(q(\mathbf{I_k})) + C \\
&= \sum_{\mathbf{I_k}\in\{0,1\}^{m_k}} q(\mathbf{I_k})\log(\frac{\widetilde{f}(\mathbf{I_k},\mathbf{y_k})}{q(\mathbf{I_k})}) + C
\end{aligned}
$$

where C is a constant unrelated with $\mathbf{I_k}$, and $\widetilde{f}(\mathbf{I_k},\mathbf{y_k})$ is

$$
\widetilde{f}(\mathbf{I_k},\mathbf{y_k}) = \exp(\int_{-\infty}^{\infty}\int_{0}^{1} q(\mu_{k1})q(p_k)\log(f(p_k,\mu_{k1},\mathbf{I_k},\mathbf{y_k}))dp_k d\mu_{k1})
$$

Using Jensen inequation we can get

$$
\begin{aligned}
&\sum_{I_{ki}\in\{0,1\}} q(\mathbf{I_k})\log(\frac{\widetilde{f}(\mathbf{I_k},\mathbf{y_k})}{q(\mathbf{I_k})}) \\
\leq\ & \log(\sum_{\mathbf{I_k}\in\{0,1\}} q(\mathbf{I_k})\frac{\widetilde{f}(\mathbf{I_k},\mathbf{y_k})}{q(\mathbf{I_k})}) \\
=\ & \log(\widetilde{f}(\mathbf{y_k}))
\end{aligned}
$$

When $q(\mathbf{I_k}) \propto \widetilde{f}(\mathbf{I_k}, \mathbf{y_k})$, $L(q)$ can be maximized. Substituting formula (4) into $\widetilde{f}(\mathbf{I_k}, \mathbf{y_k})$ we got

$$\begin{aligned}
\log(\widetilde{f}(\mathbf{I_k}, \mathbf{y_k})) &= \int_{-\infty}^{\infty}\int_0^1 q(\mu_{k1})q(p_k)\log(f(p_k, \mu_{k1}, \mathbf{I_k}, \mathbf{y_k}))dp_k d\mu_{k1} \\
&= \int_{-\infty}^{\infty}\int_0^1 q(\mu_{k1})q(p_k)\log(f(\mathbf{y_k}|p_k, \mu_{k1}, \mathbf{I_k}))dp_k d\mu_{k1} + \int_0^1 q(p_k)\log(f(\mathbf{I_k}|p_k))dp_k \\
&\quad + \int_0^1 q(p_k)\log(f(p_k))dp_k + \int_{-\infty}^{\infty} q(\mu_{k1})\log(\hat{f}(\mu_{k1}))d\mu_{k1} \\
&= \int_{-\infty}^{\infty}\int_0^1 q(\mu_{k1})q(p_k)\log(f(\mathbf{y_k}|p_k, \mu_{k1}, \mathbf{I_k}))dp_k d\mu_{k1} \\
&\quad + \int_0^1 q(p_k)\log(f(\mathbf{I_k}|p_k))dp_k + C \\
&= \int_{-\infty}^{\infty} q(\mu_{k1})\log(f(\mathbf{y_k}|\mu_{k1}, \mathbf{I_k}))d\mu_{k1} + \int_0^1 q(p_k)\log(f(\mathbf{I_k}|p_k))dp_k + C \\
&= \sum_{i=1}^{m_k}\int_{-\infty}^{\infty} q(\mu_{k1})\log(f(\mathbf{y_{ki}}|\mu_{k1}, I_{ki}))d\mu_{k1} + \sum_{i=1}^{m_k}\int_0^1 q(p_k)\log(f(I_{ki}|p_k))dp_k
\end{aligned}$$

Where $\log(f(p_k)) = 0$ because $f(p_k) = U(0,1)$. By substituting expression of $f(\mathbf{y_{ki}}|\mu_{k1}, I_{ki})$ (formula (5)) and $f(I_{ki}|p_k)$ (formula (7)), we got

$$\begin{aligned}
\log(\widetilde{f}(\mathbf{I_k}, \mathbf{y_k})) &= \sum_{i=1}^{m_k}\Big((1-I_{ki})\log(N(\mathbf{y_{ki}}|\hat{\mu}_{k0}, \hat{\sigma}_{k0}^2)) + I_{ki}\int_{-\infty}^{\infty} q(\mu_{k1})\log(N(\mathbf{y_{ki}}|\mu_{k1}, \hat{\sigma}_{k0}^2))d\mu_{k1}\Big) \\
&\quad + \sum_{i=1}^{m_k}\Big((1-I_{ki})\int_0^1 q(p_k)\log(1-p_k)dp_k + I_{ki}\int_0^1 q(p_k)\log(p_k)dp_k\Big) \\
&= \sum_{i=1}^{m_k}\Big((1-I_{ki})\big(\log(N(\mathbf{y_{ki}}|\mu_{k0}, \sigma_{k0}^2)) + \int_0^1 q(p_k)\log(1-p_k)dp_k\big) \\
&\quad + I_{ki}\big(\int_{-\infty}^{\infty} q(\mu_{k1})\log(N(\mathbf{y_{ki}}|\mu_{k1}, \hat{\sigma}_{k0}^2))d\mu_{k1} + \int_0^1 q(p_k)\log(p_k)dp_k\big)\Big)
\end{aligned}$$

and

$$\widetilde{f}(\mathbf{I_k}, \mathbf{y_k}) = \prod_{i=1}^{m_k} \rho_{ki0}^{1-I_{ki}} \rho_{ki1}^{I_{ki}}$$

where

$$\rho_{ki0} = \exp\Big(\log(N(\mathbf{y_{ki}}|\mu_{k0}, \sigma_{k0}^2)) + \int_0^1 q(p_k) \log(1-p_k) dp_k\Big) \quad (11)$$

$$\begin{aligned} \rho_{ki1} &= \exp\Big(\int_{-\infty}^{\infty} q(\mu_{k1}) \log(N(\mathbf{y_{ki}}|\mu_{k1}, \hat{\sigma}_{k0}^2)) d\mu_{k1} \\ &\quad + \int_0^1 q(p_k) \log(p_k) dp_k\Big) \end{aligned} \quad (12)$$

Because $q(\mathbf{I_k}) \propto \widetilde{f}(\mathbf{I_k}, \mathbf{y_k})$, $q(\mathbf{I_k})$ follows Bernoulli distribution and its probability density function is

$$\begin{aligned} q(\mathbf{I_k}) &= \prod_{i=1}^{m_k} q(I_{ki}) \\ &= \prod_{i=1}^{m_k} \gamma_{ki0}^{1-I_{ki}} \gamma_{ki1}^{I_{ki}} \end{aligned} \quad (13)$$

where

$$\begin{aligned} \gamma_{ki0} &= \frac{\rho_{ki0}}{\rho_{ki0} + \rho_{ki1}} \\ \gamma_{ki1} &= \frac{\rho_{ki1}}{\rho_{ki0} + \rho_{ki1}} \end{aligned}$$

Estimating $q(p_k)$ Fixing $q(\mathbf{I_k})$ and $q(\mu_{k1})$, we got

$$\begin{aligned} L(q) &= \int_0^1 q(p_k)\log(\widetilde{f}(p_k,\mathbf{y_k})) - \int_0^1 q(p_k)\log(q(p_k)) + C \\ &= \int_0^1 q(p_k)\log(\frac{\widetilde{f}(p_k,\mathbf{y_k})}{q(p_k)}) + C \end{aligned}$$

where C is a constant unrelated to p_k, and $\widetilde{f}(p_k,\mathbf{y_k})$ is

$$\widetilde{f}(p_k,\mathbf{y_k}) \; = \exp(\textstyle\sum_{\mathbf{I_k}\in\{0,1\}^{m_k}} \int_{-\infty}^{\infty} q(\mu_{k1})q(\mathbf{I_k})\log(f(p_k,\mu_{k1},\mathbf{I_k},\mathbf{y_k}))d\mu_{k1})$$

Similarly, according to Jensen inequation, $L(q)$ is maximized when $q(p_k) \propto \widetilde{f}(p_k,\mathbf{y_k})$. Substituting (4) into $\widetilde{f}(p_k,\mathbf{y_k})$ we got

$$\begin{aligned} \log(\widetilde{f}(p_k,\mathbf{y_k})) &= \sum_{\mathbf{I_k}\in\{0,1\}^{m_k}} \int_{-\infty}^{\infty} q(\mu_{k1})q(\mathbf{I_k})\log(f(p_k,\mu_{k1},\mathbf{I_k},\mathbf{y_k}))d\mu_{k1} \\ &= \sum_{\mathbf{I_k}\in\{0,1\}^{m_k}} \int_{-\infty}^{\infty} q(\mu_{k1})q(\mathbf{I_k})\log(f(\mathbf{y_k}|p_k,\mu_{k1},\mathbf{I_k}))d\mu_{k1} \\ &\quad + \sum_{\mathbf{I_k}\in\{0,1\}^{m_k}} q(\mathbf{I_k})\log(f(\mathbf{I_k}|p_k)) + q(p_k)\log(f(p_k)) \\ &= \sum_{\mathbf{I_k}\in\{0,1\}^{m_k}} \int_{-\infty}^{\infty} q(\mu_{k1})q(\mathbf{I_k})\log(f(\mathbf{y_k}|\mu_{k1},\mathbf{I_k}))d\mu_{k1} \\ &\quad + \sum_{\mathbf{I_k}\in\{0,1\}^{m_k}} q(\mathbf{I_k})\log(f(\mathbf{I_k}|p_k)) \\ &= \sum_{\mathbf{I_k}\in\{0,1\}^{m_k}} q(\mathbf{I_k})\log(f(\mathbf{I_k}|p_k)) + C \\ &= \sum_{i=1}^{m_k} \sum_{\mathbf{I_k}\in\{0,1\}^{m_k}} q(\mathbf{I_k})\log(f(I_{ki}|p_k)) + C \\ &= \sum_{i=1}^{m_k} \sum_{I_{ki}\in\{0,1\}} q(I_{ki})\log(f(I_{ki}|p_k)) + C \end{aligned}$$

where C is a constant unrelated to p_k. Substituting expression of $q(I_{ki})$ and $f(I_{ki}|p_k)$ (formula (7) and (13)) into $\log(\widetilde{f}(\mathbf{I_k}, \mathbf{y_k}))$ we get

$$\begin{aligned}\log(\widetilde{f}(p_k, \mathbf{y_k})) &= \sum_{i=1}^{m_k}\Big(\sum_{I_{ki}\in\{0,1\}} q(I_{ki})(1-I_{ki})\log(1-p_k) + \sum_{I_{ki}\in\{0,1\}} q(I_{ki}) I_{ki}\log(p_k)\Big) + C \\ &= \sum_{i=1}^{m_k}\big(\gamma_{ki0}\log(1-p_k) + \gamma_{ki1}\log(p_k)\big) + C \\ &= N_{k0}\log(1-p_k) + N_{k1}\log(p_k) + C\end{aligned}$$

where N_{k0} and N_{k1} are

$$N_{k0} = \sum_{i=1}^{m_k}\gamma_{ki0} \qquad (14)$$

$$N_{k1} = \sum_{i=1}^{m_k}\gamma_{ki1} \qquad (15)$$

As $q(p_k) \propto \widetilde{f}(p_k, \mathbf{y_k})$, $q(p_k)$ is

$$q(p_k) \propto (1-p_k)^{N_{k0}} p_k^{N_{k1}}$$

$(1-p_k)^{N_{k0}} p_k^{N_{k1}}$ is the probability density function of *Beta* distribution without normalizing constant. By normalizing it to make $\int_0^1 q(p_k)dp_k = 1$, we got

$$q(p_k) = \frac{\Gamma(N_{k0}+N_{k1}+2)}{\Gamma(N_{k0}+1)\Gamma(N_{k1}+1)}(1-p_k)^{(N_{k0}+1)-1} p_k^{(N_{k1}+1)-1} \qquad (16)$$

We used MAP of $q(p_k)$, $\hat{p}_k = \arg\max_{p_k} q(p_k)$, as point estimator of p_k, which is

$$\hat{p}_k = \frac{N_{k1}}{N_{k0}+N_{k1}}$$

Substituting expression of $q(p_k)$ (formula (16) into expression of ρ_{ki0} and ρ_{ki1} (formulas (11) and (12)) we got

$$\rho_{ki0} = \exp\Big(\log(N(\mathbf{y_{ki}}|\hat{\mu}_{k0}, \hat{\sigma}^2_{k0})) + \psi(N_{k0}+1) - \psi(N_{k0}+N_{k1}+2)\Big) \quad (17)$$

$$\rho_{ki1} = \exp\Big(\int_{-\infty}^{\infty} q(\mu_{k1}) \log(N(\mathbf{y_{ki}}|\mu_{k1}, \hat{\sigma}^2_{k0}))d\mu_{k1} + \psi(N_{k1}+1) - \psi(N_{k0}+N_{k1}+2)\Big) \quad (18)$$

where $\psi(\cdot)$ is digamma function.

Estimating $q(\mu_{k1})$ Fixing $q(p_k)$ and $q(\mathbf{I_k})$, we got

$$\begin{aligned} L(q) &= \int_{-\infty}^{\infty} q(\mu_{k1}) \log(\widetilde{f}(\mu_{k1}, \mathbf{y_k})) - \int_{-\infty}^{\infty} q(\mu_{k1}) \log(q(\mu_{k1})) + C \\ &= \int_{-\infty}^{\infty} q(\mu_{k1}) \log(\frac{\widetilde{f}(\mu_{k1}, \mathbf{y_k})}{q(\mu_{k1})}) + C \end{aligned}$$

where C is a constant unrelated to μ_{k1}, and $\widetilde{f}(\mu_{k1}, \mathbf{y_k})$ is

$$\widetilde{f}(\mu_{k1}, \mathbf{y_k}) = \exp(\textstyle\sum_{\mathbf{I_k} \in \{0,1\}^{m_k}} \int_0^1 q(p_k) q(\mathbf{I_k}) \log(f(p_k, \mu_{k1}, \mathbf{I_k}, \mathbf{y_k}))dp_k)$$

Similarly, according to Jensen inequation, $L(q)$ is maximized when $q(\mu_{k1}) \propto \widetilde{f}(\mu_{k1}, \mathbf{y_k})$. Substituting formulas (4) and (13) into $\widetilde{f}(\mu_{k1}, \mathbf{y_k})$ we got

$$
\begin{aligned}
\widetilde{f}(\mu_{k1}, \mathbf{y_k}) &= \sum_{\mathbf{I_k} \in \{0,1\}^{m_k}} q(\mathbf{I_k}) \log(f(\mathbf{y_k}|\mu_{k1}, \mathbf{I_k})) + \log(\hat{f}(\mu_{k1})) \\
&= \sum_{i=1}^{m_k} \sum_{I_{ki} \in \{0,1\}} q(I_{ki}) \log(f(y_{ki}|\mu_{k1}, I_{ki})) + \log(\hat{f}(\mu_{k1})) \\
&= \sum_{i=1}^{m_k} \big(\gamma_{ki0} \log(N(y_{ki}|\hat{\mu}_{k0}, \hat{\sigma}^2_{k0})) + \gamma_{ki1} \log(N(y_{ki}|\mu_{k1}, \hat{\sigma}^2_{k0}))\big) + \log(\hat{f}(\mu_{k1})) \\
&= \sum_{i=1}^{m_k} \gamma_{ki1} \log(N(y_{ki}|\mu_{k1}, \hat{\sigma}^2_{k0})) + \log(\hat{f}(\mu_{k1}))
\end{aligned}
$$

and

$$
q(\mu_{k1}) \propto \hat{f}(\mu_{k1}) \prod_{i=1}^{m_k} N(y_{ki}|\mu_{k1}, \hat{\sigma}^2_{k0})^{\gamma_{ki1}} \tag{19}
$$

$q(\mu_{k1})$ does not have a close form, we calculated it by numerical integration according to formula (19) and normalize it to make $\int_{-\infty}^{\infty} q(\mu_{k1}) = 1$.

Maximizing $L(q)$ by iteration Combing equations (13), (16), (17), (18) and (19) we used an iterative algorithm to maximize $L(q)$ (Algorithm S1).

Algorithm S1 Variational Inference

Set initial values $q^{(0)}(p_k)$, $q^0(\mu_{k1})$, $q^{(0)}(\mathbf{I_k})$, $\hat{p}_k^{(0)} = -1$, $\hat{p}_k^{(1)} = -2$, and $t = 1$.

while $|\hat{p}_k^{(t)} - \hat{p}_k^{(t-1)}| \geq 0.0001$ **do**

Estimating $q(\mathbf{I_k})$: According to equations (17) and (18), let

$$\rho_{ki0}^{(t)} = \exp\Big(\log(N(\mathbf{y_{ki}}|\mu_{k0}, \sigma_{k0}^2)) + \psi(N_{k0}^{(t-1)} + 1) - \psi(N_{k0}^{(t-1)} + N_{k1}^{(t-1)} + 2)\Big)$$

$$\rho_{ki1}^{(t)} = \exp\Big(\int_{-\infty}^{\infty} q^{(t-1)}(\mu_{k1}) \log(N(\mathbf{y_{ki}}|\mu_{k1}, \hat{\sigma}_{k0}^2))d\mu_{k1} + \psi(N_{k1}^{(t-1)} + 1) - \psi(N_{k0}^{(t-1)} + N_{k1}^{(t-1)} + 2)\Big)$$

According to equation (13), let

$$\gamma_{ki0}^{(t)} = \frac{\rho_{ki0}^{(t)}}{\rho_{ki0}^{(t)} + \rho_{ki1}^{(t)}}$$

$$\gamma_{ki1}^{(t)} = \frac{\rho_{ki1}^{(t)}}{\rho_{ki0}^{(t)} + \rho_{ki1}^{(t)}}$$

Then we get estimated $q(\mathbf{I_k})$ in the tth step, which is

$$q^{(t)}(\mathbf{I_k}) = \prod_{i=1}^{m_k} (\gamma_{ki0}^{(t)})^{1-I_{ki}} (\gamma_{ki1}^{(t)})^{I_{ki}}$$

Estimating $q(p_k)$: According to equations (14) and (15), let

$$N_{k0}^{(t)} = \sum_{i=1}^{m_k} \gamma_{ki0}^{(t)}$$

$$N_{k1}^{(t)} = \sum_{i=1}^{m_k} \gamma_{ki1}^{(t)}$$

Then we get estimated $q(p_k)$ in the tth step, which is

$$q^{(t)}(p_k) = \frac{\Gamma(N_{k0}^{(t)} + N_{k1}^{(t)} + 2)}{\Gamma(N_{k0}^{(t)} + 1)\Gamma(N_{k1}^{(t)} + 1)} (1 - p_k)^{(N_{k0}^{(t)} + 1) - 1} p_k^{(N_{k1}^{(t)} + 1) - 1}$$

and estimated p_k at the tth step is

$$\hat{p}_k^{(t)} = \frac{N_{k1}^{(t)}}{N_{k0}^{(t)} + N_{k1}^{(t)}}$$

Estimating $q(\mu_k)$: calculate $q(\mu_k)$ numerically according to equation (19), which is

$$q^{(t)}(\mu_{k1}) \propto \hat{f}(\mu_{k1}) \prod_{i=1}^{m_k} N(y_{ki}|\mu_{k1}, \hat{\sigma}_{k0}^2)^{\gamma_{ki1}^{(t)}}$$

Set $t = t + 1$

end while

Assuming Algorithm S1 stop at the Tth step, estimated posteriori probability density function of p_k is

$$\hat{f}(p_k|\mathbf{y_k}) = \frac{\Gamma(N_{k0}^{(T)}+N_{k1}^{(T)}+2)}{\Gamma(N_{k0}^{(T)}+1)\Gamma(N_{k1}^{(T)}+1)}(1-p_k)^{(N_{k0}^{(T)}+1)-1}p_k^{(N_{k1}^{(T)}+1)-1}$$

Point estimation of p_k is

$$\hat{p}_k = \frac{N_{k1}^{(T)}}{N_{k0}^{(T)}+N_{k1}^{(T)}} \tag{20}$$

$\hat{p}_k$ is actually the ratio between estimated expectation of number of kinetic variant bases covering the kth loci and total number of bases covering the kth loci, because $N_{k0} + N_{k1} = m_k$, $N_{k1} = \sum_{i=1}^{m_k} \gamma_{ki1}$ and γ_{ki1} is estimated probability of the ith base covering the kth loci is variant (equation (13)).

Initial values To minimize impact of external information introduced, we set

$$\gamma_{ki0}^{(0)} = \frac{1}{2}$$
$$\gamma_{ki1}^{(0)} = \frac{1}{2}$$

and according to equations (14) and (15), $N_{k0}^{(0)} = \frac{m_k}{2}$ and $N_{k1}^{(0)} = \frac{m_k}{2}$. Thus $q^{(0)}(\mathbf{I_k})$ and $q^{(0)}(p_k)$ are

$$q^{(0)}(\mathbf{I_k}) = \prod_{i=1}^{m_k}(\gamma_{ki0}^{(0)})^{1-I_{ki}}(\gamma_{ki1}^{(0)})^{I_{ki}}$$
$$q^{(0)}(p_k) = \frac{\Gamma(N_{k0}^{(0)} + N_{k1}^{(0)} + 2)}{\Gamma(N_{k0}^{(0)} + 1)\Gamma(N_{k1}^{(0)} + 1)}(1-p_k)^{(N_{k0}^{(0)}+1)-1}p_k^{(N_{k1}^{(0)}+1)-1}$$

We used $\hat{f}(\mu_{k1})$ as initial value of $q(\mu_{k1})$, i.e.

$$q^{(0)}(\mu_{k1}) = \hat{f}(\mu_{k1})$$

Expectation maximization (EM) algorithm for fitting standard mixture model

As described in section **empirical Bayes mixture model** in the main text, IPD distribution of the kth locus is

$$f(\mathbf{y_k}|\mu_{k0}, \sigma_{k0}^2, \mu_{k1}, \sigma_{k1}^2, p_k) = \prod_{i=1}^{m_k} f(\mathbf{y_{ki}}|\mu_{k0}, \sigma_{k0}^2, \mu_{k1}, \sigma_{k1}^2, p_k) \tag{21}$$

and

$$\begin{aligned} f(\mathbf{y_{ki}}|\mu_{k0}, \sigma_{k0}^2, \mu_{k1}, \sigma_{k1}^2, p_k) &= (1-p_k) \cdot N(\mathbf{y_{ki}}|\mu_{k0}, \sigma_{k0}^2) + p_k \cdot N(\mathbf{y_{ki}}|\mu_{k1}, \sigma_{k1}^2) \quad (22) \\ &= (1-p_k) \cdot \prod_{j=1}^{n_{ki}} N(y_{kij}|\mu_{k0}, \sigma_{k0}^2) + p_k \cdot \prod_{j=1}^{n_{ki}} N(y_{kij}|\mu_{k1}, \sigma_{k1}^2) \end{aligned}$$

where μ_{k0}, σ_{k0} and σ_{k1} are regard as known parameters because they can be pre-estimated from WGA control data, p_k and μ_{k1} are the two unknown parameters to be estimated from data $\mathbf{y_k}$.

We used EM algorithm to estimate p_k and μ_{k1} by maximizing likelihood function $f(\mathbf{y_k}|\mu_{k0}, \sigma_{k0}^2, \mu_{k1}, \sigma_{k1}^2, p_k)$. We introduced indicator I_{ki}, where $I_{ki} = 1$ is the ith base covering the kth locus is kinetic variant, otherwise $I_{ki} = 0$, and assume its probability mass function is

$$f(I_{ki}|p_k) = (1-p_k)^{1-I_{ki}} p_k^{I_{ki}}$$

Likelihood function of full data $(\mathbf{y_k}, \mathbf{I_k})$ is

$$f(\mathbf{y_k}, \mathbf{I_k}|p_k, \mu_{k1}) = f(\mathbf{y_k}|\mathbf{I_k}, \mu_{k1}) f(\mathbf{I_k}|p_k)$$

where $\mathbf{I_k} = \{I_{k1}, I_{k2}, ..., I_{km_k}\}$, $f(\mathbf{I_k}|p_k) = \prod_{i=1}^{m_k}(1-p_k)^{1-I_{ki}}p_k^{I_{ki}}$ and

$$\begin{aligned} f(\mathbf{y_k}|\mathbf{I_k}, \mu_{k1}) &= \prod_{i=1}^{m_k} f(\mathbf{y_{ki}}|I_{ki}, \mu_{k1}) \\ &= \prod_{i=1}^{m_k} N(\mathbf{y_{ki}}|\mu_{k0}, \sigma_{k0}^2)^{1-I_{ki}} N(\mathbf{y_{ki}}|\mu_{k1}, \sigma_{k1}^2)^{I_{ki}} \end{aligned}$$

Logarithmic likelihood function of full data $(\mathbf{y_k}, \mathbf{I_k})$ is

$$\begin{aligned} \ell(p_k, \mu_{k1}|\mathbf{y_k}, \mathbf{I_k}) &= \sum_{i=1}^{m_k} \Big((1-I_{ki})\big(N(\mathbf{y_{ki}}|\mu_{k0}, \sigma_{k0}^2) + \log(1-p_k)\big) \\ &\quad + I_{ki}\big(N(\mathbf{y_{ki}}|\mu_{k1}, \sigma_{k1}^2) + \log(p_k)\big)\Big) \end{aligned}$$

E-step : at the tth step of the EM algorithm,

$$\begin{aligned} & E\big(\ell(p_k, \mu_{k1}|\mathbf{y_k}, \mathbf{I_k})|\mathbf{y_k}, p_k^{(t-1)}, \mu_{k1}^{(t-1)}\big) \\ = & \sum_{i=1}^{m_k} \Big((1-\gamma_{ki}^{(t)})\big(N(\mathbf{y_{ki}}|\mu_{k0}, \sigma_{k0}^2) + \log(1-p_k)\big) \\ & + \gamma_{ki}^{(t)}\big(N(\mathbf{y_{ki}}|\mu_{k1}, \sigma_{k1}^2) + \log(p_k)\big)\Big) \end{aligned}$$

where $p_k^{(t-1)}$, $\mu_{k1}^{(t-1)}$ are parameters estimated at $(t-1)$th step and

$$\begin{aligned} \gamma_{ki}^{(t)} &= \Pr(I_{ki} = 1|\mathbf{y_k}, p_k^{(t-1)}, \mu_{k1}^{(t-1)}) \\ &= \frac{p_k^{(t-1)} N(\mathbf{y_{ki}}|\mu_{k1}, \sigma_{k1}^2)}{p_k^{(t-1)} N(\mathbf{y_{ki}}|\mu_{k1}, \sigma_{k1}^2) + (1-p_k^{(t-1)}) N(\mathbf{y_{ki}}|\mu_{k0}, \sigma_{k0}^2)} \end{aligned}$$

M-step : $E\big(\ell(p_k, \mu_{k1}|\mathbf{y_k}, \mathbf{I_k})|\mathbf{y_k}, p_k^{(t-1)}, \mu_{k1}^{(t-1)}\big)$ can be maximized by setting p_k and μ_{k1} to be

$$
\begin{aligned}
p_k^{(t)} &= \frac{m_k - \sum_{i=1}^{m_k} \gamma_{ki}^{(t)}}{m_k} \\
\mu_{k1}^{(t)} &= \frac{\sum_{i=1}^{m_k} \gamma_{ki}^{(t)} \sum_{j=1}^{n_{ki}} y_{kij}}{\sum_{i=1}^{m_k} \gamma_{ki}^{(t)} n_{ki}}
\end{aligned}
$$

E-step and M-step are repeated until convergence. The full EM algorithm is summarized in Algorithm S2.

Algorithm S2 Expectation maximization algorithm

Set initial values of μ_{k1} to be $\mu_{k1}^{(0)}$. Set $t = 1, p_k^{(0)} = -1, p_k^{(1)} = -2$.

while $|p_k^{(t)} - p_k^{(t-1)}| \geq 0.0001$ **do**

Step 1: calculate $\gamma_{ki}^{(t)}$ according to

$$
\gamma_{ki}^{(t)} = \frac{p_k^{(t-1)} N(\mathbf{y_{ki}}|\mu_{k1}^{(t-1)}, \sigma_{k1}^2)}{p_k^{(t-1)} N(\mathbf{y_{ki}}|\mu_{k1}^{(t-1)}, \sigma_{k1}^2) + (1 - p_k^{(t-1)}) N(\mathbf{y_{ki}}|\mu_{k0}, \sigma_{k0}^2)}
$$

Step 2: calculate $p_k^{(t)}$ according to

$$
p_k^{(t)} = \frac{m_k - \sum_{i=1}^{m_k} \gamma_{ki}^{(t)}}{m_k}
$$

Step 3: calculate $\mu_{k1}^{(t)}$ according to

$$
\mu_{ki}^{(t)} = \frac{\sum_{i=1}^{m_k} \gamma_{ki}^{(t)} \sum_{j=1}^{n_{ki}} y_{kij}}{\sum_{i=1}^{m_k} \gamma_{ki}^{(t)} n_{ki}}
$$

Set $t = t + 1$

end while

Initial values The only initial value needed to be determined in Algorithm S2 is $\mu_{k1}^{(0)}$. To avoid the local optimal problem of EM algorithm, we tried 12 initial values: $\mu_{k1}^{(0)} = 0.25$, $\mu_{k1}^{(0)} = 0.5$, $\mu_{k1}^{(0)} = 0.75$,..., $\mu_{k1}^{(0)} = 3$ to fit the model and selected the

result that has the largest likelihood function $f(\mathbf{y_k}|\mu_{k0}, \sigma^2_{k0}, \mu_{k1}, \sigma^2_{k1}, p_k)$ as the final result.

References

[1] Efron,B. (2004) Large-scale simultaneous hypothesis testing: the choice of a null hypothesis. *Jour Amer Stat Assoc*, **99**, 96-104.

[2] Efron,B. (2007) Correlation and Large-Scale Simultaneous Sinificance Testing. *Jour Amer Stat Assoc*, **102**, 93-104.
