# Supplementary material for: qDNAmod: a statistical model-based tool to reveal intercellular heterogeneity of DNA modification from SMRT sequencing data
Source: Nucleic Acids Res. 2014 Nov 17;42(22):13488–99. doi: 10.1093/nar/gku1097 (PMC4267614; doi:10.1093/nar/gku1097)
Supplement: SUPPLEMENTARY DATA [file supp_gku1097_nar-02867-z-2014-File005.zip › Supplementary_Data/Table_S1-3.docx]

**Table S1. Bacterial strains used in this study**

| **Strain** | **Description** | **Source or reference** |
| --- | --- | --- |
| ER2796 | *E. coli* derivative, a strain lacking endogenous methyltransferase activities; Tet^r^; Kan^r^ | (1) |
| ST556 | *S. pneumoniae,* serotype 19F, a multidrug-resistant isolate from a patient with otitis media; Cm^s^; Kan^s^ | (2) |
| TH4992 | ST556 derivative; △MYY0570-MYY0572::Janus cassette; Kan^r^ | This study |
| TH4993 | ST556 derivative; △MYY0859-MYY0860::Janus cassette; Kan^r^ | This study |
| TH4994 | ST556 derivative; ST556 transformed with pTH4994; Cm^r^, Kan^r^ | This study |
| TH4995 | ST556 derivative; ST556 transformed with pTH4995; Cm^r^, Kan^r^ | This study |
| TH4996 | ST556 derivative; ST556 transformed with pTH4996; Cm^r^, Kan^r^ | This study |
| TH4997 | ST556 derivative; ST556 transformed with pTH4997; Cm^r^, Kan^r^ | This study |
| TH4998 | ST556 derivative; ST556 transformed with pTH4998; Cm^r^, Kan^r^ | This study |
| TH4999 | TH4992 derivative; TH4992 transformed with pTH4994; Cm^r^, Kan^r^ | This study |
| TH5000 | TH4992 derivative; TH4992 transformed with pTH4995; Cm^r^, Kan^r^ | This study |
| TH5001 | TH4992 derivative; TH4992 transformed with pTH4996; Cm^r^, Kan^r^ | This study |
| TH5002 | TH4992 derivative; TH4992 transformed with pTH4997; Cm^r^, Kan^r^ | This study |
| TH5003 | TH4993 derivative; TH4993 transformed with pTH4998; Cm^r^, Kan^r^ | This study |

Tet: tetracycline; Kan: kanamycin; Cm: chloramphenicol

r: resistant; s: sensitive

**Table S2. Plasmids used in this study**

| **Plasmids** | **Description** | **Source or reference** |
| --- | --- | --- |
| pRRS | A vector for expressing methyltransferase genes; Ap^r^ | (1) |
| pTH4832 | pRRS::MYY0571-MYY0570; coding sequence of MYY0571 and MYY0570 cloned in PstI/BamHI sites of pRRS followed by 5’-AAGTACTTTTTTTTG-3’; Ap^r^ | This study |
| pTH4833 | pRRS::MYY0571-MYY0570; coding sequence of MYY0571 and MYY0570 cloned in PstI/BamHI sites of pRRS followed by 5’-AAGTACTTTTTTTCG-3’; Ap^r^ | This study |
| pTH4834 | pRRS::MYY0859; coding sequence of MYY0859 cloned in PstI/BamHI sites of pRRS followed by 5’-TCTAGA-3’; Ap^r^ | This study |
| pTH4835 | pRRS::MYY1312-MYY1311; coding sequence of MYY1312 and MYY1311 cloned in PstI/BamHI sites of pRRS followed by 5’- TGACTAGTAATATC-3’; Ap^r^ | This study |
| pTH4836 | pRRS::frame-shifted MYY0571-MYY0570 followed by 5’-AAGTACTTTTTTTTG-3’; an adenine was inserted in the site following the start codon of MYY0571; Ap^r^ | This study |
| pTH4837 | pRRS::frame-shifted MYY0571-MYY0570 followed by 5’-AAGTACTTTTTTTCG-3’; an adenine was inserted in the site following the start codon of MYY0571; Ap^r^ | This study |
| pTH4838 | pRRS::frame-shifted MYY0859; followed by 5’-TCTAGA-3’; an adenine was inserted in the site following the start codon of MYY0859; Ap^r^ | This study |
| pTH4839 | pRRS::frame-shifted MYY1312-MYY1311; followed by 5’-TGACTAGTAATATC-3’; an adenine was inserted in the site following the start codon of MYY1312; Ap^r^ | This study |
| pST393 | 248 base pairs coding sequence of *hk06* in *S. pneumoniae* R6 strain in SmaI site of pID701t; Cm^r^ | (3) |
| pTH4994 | 5’-AAGTACTTCGGTTTG-3’ cloned in the site following the stop codon of chloramphenicol resistant gene of pST393; Cm^r^ | This study |
| pTH4995 | 5’-AAGTACTTTTTTTTG-3’ cloned in the site following the stop codon of chloramphenicol resistant gene of pST393; Cm^r^ | This study |
| pTH4996 | 5’-AAGTACTTGAGTTCG-3’ cloned in the site following the stop codon of chloramphenicol resistant gene of pST393; Cm^r^ | This study |
| pTH4997 | 5’-AAGTACTTTTTTTCG-3’ cloned in the site following the stop codon of chloramphenicol resistant gene of pST393; Cm^r^ | This study |
| pTH4998 | 5’-TCTAGA-3’ cloned in the site following the stop codon of chloramphenicol resistant gene of pST393; Cm^r^ | This study |

Ap^r^: carrying β lactamase gene; Cm^r^: carrying chloramphenicol acetyltransferase gene;

**Table S3. Primers used in this study**

| **Number** | **Sequencing** |
| --- | --- |
| Pr1097 | 5’-GAGATCTAGACCGTTTGATTTTTAATGGATAATG-3’ |
| Pr1098 | 5’-GAGACTCGAGCCTTTCCTTATGCTTTTGGAC-3' |
| Pr6929 | 5’-GAGACTCGAGATTGAACCCTCCGC-3’ |
| Pr6930 | 5’-CAAACTCGTACAAACAACAAGACCACCTAA-3’ |
| Pr6931 | 5’-GAGATCTAGATTCTTTTCATCTCTTCATGATTAGA-3’ |
| Pr6932 | 5’-GAACTTAAAGAGTATTTGATAGCTAGTACAGCATTTATTG-3’ |
| Pr6941 | 5'-GAGATCTAGATTTGCAAATTATTTACAGAAAGAACA-3' |
| Pr6942 | 5'-AGAAGATAGGATAGATATTTCTCACTATTTAGTTGATGAC-3' |
| Pr6943 | 5'-GTGTCTCGAGTTCACGAACTCTCTCTTG-3' |
| Pr6944 | 5'-GGCACTGGCTTGGGTAGAAAGGT-3' |
| Pr6991 | 5’-GTTCTGCAGTTAAGGTTAATCATATGACAGTATTAAAAGGAGA-3’ |
| Pr6992 | 5’-GTTGGATCCTCTAGACTATAGTAACTGTTGACTTTTACTTAACCAT-3’ |
| Pr6993 | 5’-GTTCTGCAGTTAAGGTTAATCATATGTCAATTACATCATTTGTAAAAAG-3’ |
| Pr6994 | 5’-GTTGGATCCGATATTAAGCTTCAtcatttccaaagttgattta-3’ |
| Pr7094 | 5’-GTTCTGCAGTTAAGGTTAATCATatgattacaggcgaattaaaaaata-3’ |
| Pr7095 | 5’-GTTGGATCCCAGTTTACTAGTGTCAGCCAAAATACTCCTGCATCAGAGATTT-3’ |
| Pr7102 | 5’-TGACTAGTAATATCGGATCCCCGGGGAAGATCTAGATCTAGATA-3’ |
| Pr7109 | 5’-GATATTACTAGTCATCAGCCAAAATACTCCTGCATCAGAGATTT-3’ |
| Pr7389 | 5’-aagtactTTTTtttgGGATCCCCGGGGAAGATCTAGATCTAGATA-3’ |
| Pr7390 | 5’-aagtactTTTTttcgGGATCCCCGGGGAAGATCTAGATCTAGATA-3’ |
| Pr7393 | 5’-caaaAAAAagtacttTCATTTCCAAAGTTGATTTACTTTTTCAAA-3’ |
| Pr7395 | 5’-cgaaAAAAagtacttTCATTTCCAAAGTTGATTTACTTTTTCAAA-3’ |
| Pr7653 | 5’-CCCTGCATTTATTTTCTTAGTGACAAGGGTGATAA-3’ |
| Pr7654 | 5’-ATAAAAGCCAGTCATTAGGCCTATCTGACAATTCC-3’ |
| Pr7742 | 5’-AAGGTTAATCATatgatcaattacatcattt-3’ |
| Pr7743 | 5’-aaatgatgtaattgatcatATGATTAACCTT-3’ |
| Pr7744 | 5’-AAGGTTAATCATatgaacagtattaaaagga-3’ |
| Pr7745 | 5’-tccttttaatactgttcatATGATTAACCTT-3’ |
| Pr7746 | 5’-AAGGTTAATCATatgaattacaggcgaatta-3’ |
| Pr7747 | 5’-taattcgcctgtaattcatATGATTAACCTT-3’ |
| Pr7771 | 5’-ATCTAGATTTTTATCTTTTACAGTCGGTTTTCTAATGTCACTAA-3’ |
| Pr7772 | 5-GATAAAAATCTAGATAGTACAGTCGGCATTATCTCATATTATAAAA-3’ |
| Pr7773 | 5’-AAGTACTTCGGTTTGTTTTACAGTCGGTTTTCTAATGTCACTAA-3’ |
| Pr7774 | 5’-CAAACCGAAGTACTTAGTACAGTCGGCATTATCTCATATTATAAAA-3’ |
| Pr7775 | 5’-AAGTACTTTTTTTTGTTTTACAGTCGGTTTTCTAATGTCACTAA-3’ |
| Pr7776 | 5’-CAAAAAAAAGTACTTAGTACAGTCGGCATTATCTCATATTATAAAA-3’ |
| Pr7777 | 5’-AAGTACTTGAGTTCGTTTTACAGTCGGTTTTCTAATGTCACTAA-3’ |
| Pr7778 | 5’-CGAACTCAAGTACTTAGTACAGTCGGCATTATCTCATATTATAAAA-3’ |
| Pr7779 | 5’-AAGTACTTTTTTTCGTTTTACAGTCGGTTTTCTAATGTCACTAA-3’ |
| Pr7780 | 5’-CGAAAAAAAGTACTTAGTACAGTCGGCATTATCTCATATTATAAAA-3’ |
| Pr7797 | 5’-GTTAAGGTTAATCATATGTCAATTACATCATTTGTAAA-3’ |
| Pr7798 | 5’-TTTACAAATGATGTAATTGACATATGATTAACCTTAAC-3’ |
| Pr7799 | 5’-GTTAAGGTTAATCATATGACAGTATTAAAAGGAGATAACT-3’ |
| Pr7800 | 5’-AGTTATCTCCTTTTAATACTGTCATATGATTAACCTTAAC-3’ |
| Pr7801 | 5’-GTTAAGGTTAATCATATGATTACAGGCGAATTAA-3’ |
| Pr7802 | 5’-TTAATTCGCCTGTAATCATATGATTAACCTTAAC-3’ |

**References**

1. **Murray, I. A., T. A. Clark, R. D. Morgan, M. Boitano, B. P. Anton, K. Luong, A. Fomenkov, S. W. Turner, J. Korlach, and R. J. Roberts.** 2012. The methylomes of six bacteria. Nucleic Acids Res **40:**11450-62.

2. **Chen, H., Y. Ma, J. Yang, C. J. O'Brien, S. L. Lee, J. E. Mazurkiewicz, S. Haataja, J. H. Yan, G. F. Gao, and J. R. Zhang.** 2008. Genetic requirement for pneumococcal ear infection. PLoS One **3:**e2950.

3. **Ma, Z., and J. R. Zhang.** 2007. RR06 activates transcription of spr1996 and cbpA in Streptococcus pneumoniae. J Bacteriol **189:**2497-509.
